# New insight into the taxonomy of Cephaloziellaceae (Marchantiophyta): the family of the smallest higher plants on Earth

**DOI:** 10.3389/fpls.2024.1326810

**Published:** 2024-02-29

**Authors:** Vadim A. Bakalin, Yulia D. Maltseva, Ksenia G. Klimova, Van Sinh Nguyen, Seung Se Choi, Aleksey V. Troitsky

**Affiliations:** ^1^ Laboratory of Cryptogamic Biota, Botanical Garden-Institute Far Eastern Branch of the Russian Academy of Sciences (FEB RAS), Vladivostok, Russia; ^2^ Department of Plant Ecology, Institute of Ecology and Biological Resources, Graduate University of Science and Technology, Vietnam Academy of Science and Technology, Ha Noi, Vietnam; ^3^ Team of National Ecosystem Survey, National Institute of Ecology, Seocheon, Republic of Korea; ^4^ Belozersky Institute of Physico-Chemical Biology, Lomonosov Moscow State University, Moscow, Russia

**Keywords:** *Cephaloziella*, *Cephaloziopsis*, ITS1–2, *Metacephalozia*, *psb*A, *rbc*L, *trn*G, *trn*L–F

## Abstract

An analysis of the phylogeny of Cephaloziellaceae was carried out based on trees constructed for previously and newly obtained sequences of five genes: nuclear ITS1–2 and chloroplast *trn*L–F, *trn*G, *rbc*L, and *psb*A. Phylogenetic trees inferred from different genes are congruent for the main details; however, the position of several taxa is variable. As a result, a new phylogenetic system of the family was proposed. The narrow genus concept seems to be more appropriate for the family. *Cephaloziella spinicaulis* is segregated into the new genus *Douiniella*, the generic status for *Prionolobus* and *Metacephalozia* is confirmed, and the dubious generic status of *Kymatocalyx* is substantiated. The generic independence of *Cylindrocolea* from *Cephaloziella* s. str. is confirmed. The small amount of data hinders the description of two more genera from *Cephaloziella* s.l.

## 1 Introduction

Cephaloziellaceae plants are the smallest among higher plants. Despite sometimes not reaching a size of 1 mm, they are well developed and have reproductive organs. For a long time, this led to subjective difficulties in comprehending the taxonomy of the family: Cephaloziellaceae are commonly overlooked in field exploration, and when studied in the laboratory, morphological features that delimit species are often difficult to recognize. Despite microscopes now being much better than they were 100 years ago and before, these difficulties, to a certain extent, still challenge researchers who study this group of plants.

Montagne (1856) was the first to describe the genus *Dichiton* Mont. in the suite of currently recognized Cephaloziellaceae. The genus was forgotten for a long time and remembered when the generic name *Cephaloziella* (Spruce) Schiffn. became widely known. Moreover, the number of known species was so significant that, despite the earlier appearance, the name *Cephaloziella* was assigned *nom. cons*. with priority over *Dichiton* published 40 years before. The purposeful study of *Cephaloziella* began with Spruce (1882), who assigned the taxonomic status of this group of plants to *Cephalozia* subg. *Cephaloziella* Spruce and subg. *Prionolobus* Spruce (Spruce, 1882). After 11 years, this subgenus was elevated to the rank of genus by Schiffner (1893): *Cephaloziella* (Schiffner, 1893). Schiffner also recognized *Prionolobus* (Spruce) Schiffn., which is actually the evaluated *Cephalozia* subg. *Prionolobus*.

A remarkable new step in the knowledge of Cephaloziellaceae was made by Douin, who segregated this family (Douin, 1920) and perfectly described the features differentiating Cephaloziaceae and Cephaloziellaceae. In addition, he also accepted (described or re-evaluated) the following genera within Cephaloziellaceae: *Dichiton*, *Lophoziella* Douin (where the type species is the same as that of *Dichiton*), *Prionolobus*, *Evansia* Douin et Schiffn. (illegitimate name, later homonym of *Evansia* Salisb. ex Decne.), *Cephaloziella* and *Protocephaloziella* Douin. The next major contribution to the knowledge of Cephaloziellaceae taxonomy was made by Schuster (several works, with the fundamental one published in 1972) (Schuster, 1972). Schuster described the following genera: *Amphicephalozia* R.M.Schust., *Cephalomitrion* R.M.Schust., *Cylindrocolea* R.M.Schust., and *Gymnocoleopsis* (R.M.Schust.) R.M.Schust. The latest World Checklist of 2016 includes 171 accepted Cephaloziellaceae species names from 19 genera (Söderström et al., 2016).

All authors of the 19th and 20th centuries regarded *Cephaloziella* as closely related to *Cephalozia* (Dumort.) Dumort. However, recent molecular phylogenies (De Roo et al., 2007; Vilnet et al., 2012; Patzak et al., 2016; Bakalin et al., 2021a) have shown that these genera are not related; in contrast, Cephaloziellaceae were found to be genetically more similar to Lophoziaceae Cavers, Scapaniaceae Mig., and Anastrophyllaceae L.Söderstr., commonly showing morphologies unlike the Cephaloziellaceae “archetype” morphology.

Although several robust studies have been carried out, knowledge of intrafamilial taxonomy, ecology, and distribution of Cephaloziellaceae is limited compared with that of many other liverwort families. Moreover, it is noteworthy that the onset of the “molecular era” contributed relatively little to the creation of a clear taxonomic system for the family, and the main reason for this, as best we can infer, is still the same as 100 years ago: the small size of the plants and their common growth in mixtures (often as very scarce admixtures) with other bryophytes make it difficult to collect samples for molecular analysis. The new materials that we obtained during the study of mostly Indochinese liverworts allowed us to shed some light on the taxonomy of Cephaloziellaceae. Moreover, having initially become interested in the study of *Cephaloziopsis* (Spruce) Schiffn., as a result of our efforts, the system of Cephaloziellaceae was proposed to be revised on the basis of new molecular data as much as possible using the materials and data available to us. The presentation of the results obtained is the goal of this work.

## 2 Materials and methods

### 2.1 Taxon sampling

To compile our dataset for molecular phylogenetic analysis, species from the suborder Cephaloziineae Schljakov (Mamontov and Vilnet, 2017) were sampled. Species of *Hygrobiella* Spruce–*Hygrobiella laxifolia* (Hook.) Spruce (for *rbc*L and *psb*A), *Hygrobiella nishimurae* N.Kitag., and *Hygrobiella squamosa* Bakalin et Vilnet (for ITS1–2, *trn*L–F, and *trn*G) (Hygrobiellaceae and Jungermanniineae) were selected as an outgroup (Feldberg et al., 2013). In total, 141 species were included in the analysis, one to three species of *Hygrobiella* (depending on sequences availability) are in outgroups, and others are in ingroups (**Supplementary Table 1**); sequences of 15 species from the families Cephaloziellaceae and Hygrobiellaceae (5 ITS1–2, 15 *trn*L–F, 15 *trn*G, 7 *rbc*L, and 8 *psb*A) were obtained by the authors, and nucleotide data for 126 specimens were downloaded from the National Center for Biotechnology Information (NCBI) GenBank. DNA vouchers, including GenBank accession numbers and voucher details, are listed in **Supplementary Table 1**. The selection of genera involved in the study is identified by the goal of the paper described in the Introduction section (including a study of the taxonomic position of *Cephaloziopsis exigua*), as well as the presence of data on Cephaloziellaceae s. str. in the GenBank. All Cephaloziellaceae materials available in the GenBank for involved loci were used in this account preparation. Furthermore, in addition to Cephaloziellaceae in the narrow sense, we were involved in the study of related genera belonging to the same superclade Cephaloziellaceae–Scapaniaceae and forming the core of the phylogenetic system of the suborder Cephaloziineae. The involvement of a certain number of genera outside of Cephaloziellaceae s. str. made it possible to more reliably reveal phylogenetic relationships within Cephaloziellaceae and provide a more accurate circumscription of the family in a narrow sense.

### 2.2 DNA isolation, amplification, and sequencing

DNA was extracted from dried liverwort tissues using the NucleoSpin Plant II Kit (Macherey-Nagel, Düren, Germany) and DNeasy Plant Mini Kit (Qiagen, Hilden, Germany) following the manufacturers’ protocols. Amplification of ITS1–2, *trn*L–F, *trn*G-intron, *rbc*L, and *psb*A was performed using an Encyclo Plus PCR Kit (Evrogen, Moscow, Russia) with the primers listed in **Table 1**.

**Table 1:** Primers used in polymerase chain reaction (PCR) and cycle sequencing.

Locus	Sequence (5′–3′)	Direction	Annealing temperature (°C)	Reference
ITS1–2 nrDNA	CGGTTCGCCGCCGGTGACG	Forward	68	Groth et al., 2002
ITS1–2 nrDNA	ACCTGCGGAAGGATCATTG	Forward	58	Friedl, 1996
ITS1–2 nrDNA	GATATGCTTAAACTCAGCGG	Reverse	58	Milyutina et al., 2010
*trn*L–F cpDNA	CGAATTCGGTAGACGCTACG	Forward	62	Taberlet et al., 1991
*trn*L–F cpDNA	CGAAATTGGTAGACGCTGCG	Forward	62	Bakalin et al., 2021b
*trn*L–F cpDNA	ATTTGAACTGGTGACACGAG	Reverse	58	Taberlet et al., 1991
*trn*L–F cpDNA	TGCCAGAAACCAGATTTGAAC	Reverse	58	Bakalin et al., 2021b
*trn*G-intron cpDNA	ACCCGCATCGTTAGCTTG	Forward	56	Pacak and Szweykowska-Kulińska, 2000
*trn*G-intron cpDNA	GCGGGTATAGTTTAGTGG	Reverse	54	Pacak and Szweykowska-Kulińska, 2000
*rbc*L	ATGTCACCACAAACGGA	Forward	50	Fedosov et al., 2016
*rbc*L	TCAAATTCAAACTTGATTTCTTTCCA	Reverse	66	Fedosov et al., 2016
*psb*A	TTTCTCAGACGGTATGCC	Forward	54	Cox et al., 2000
*psb*A	GACGAGTTCCGGGTTCGA	Forward	58	Cox et al., 2000
*psb*A	TGGAATGGGTGCATAAGG	Reverse	54	Cox et al., 2000
*psb*A	GAACGACGGGAATTGAAC	Reverse	54	Cox et al., 2000

Polymerase chain reaction was performed in a total volume of 20 µl, including 1 µl of template DNA, 0.4 µl of Encyclo polymerase, 5 µl of Encyclo buffer, 0.4 µl of dNTP mixture (included in the Encyclo Plus PCR Kit), 13.4 µl (for *trn*L–F, *trn*G-intron, *rbc*L, and *psb*A)/12.4 µl (for ITS1–2) of double-distilled water (Evrogen, Moscow, Russia), 1 µl of dimethylsulfoxide (for ITS1–2), and 0.4 µl of each primer (forward and reverse, at a concentration of 5 pmol/µl).

Polymerase chain reactions were carried out using the following program:


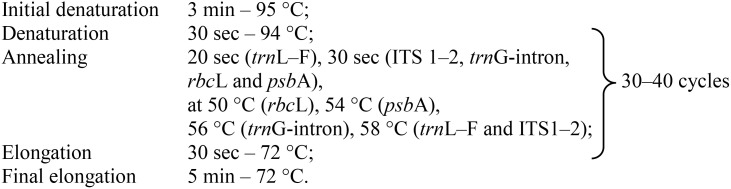


Amplified fragments were visualized after electrophoresis on 1% agarose TAE gels by EthBr staining and purified using the Cleanup Mini Kit (Evrogen, Moscow, Russia). The DNA was sequenced using the ABI PRISM^®^ BigDye™ Terminator Cycle Sequencing Ready Reaction Kit (Applied Biosystems, Foster City, CA, USA) with further analysis of the reaction products following the standard protocol on an automatic sequencer (3730 DNA Analyzer, Applied Biosystems, USA) at the Genome Center (Engelhardt Institute of Molecular Biology, Russian Academy of Sciences, Moscow, Russia).

### 2.3 Phylogenetic analyses

The datasets were produced for the ITS1–2, *trn*L–F, *trn*G-intron, *rbc*L, and *psb*A loci. All sequences were aligned using MAFFT (Katoh and Standley, 2013) with standard settings and then edited manually in BioEdit ver. 7.2.5 (Hall, 1999). All positions of the final alignments were included in the phylogenetic analyses. Phylogenies were reconstructed under two criteria: maximum likelihood (ML) with IQ-tree ver. 1.6.12 (Nguyen et al., 2015) and Bayesian inference (BI) with MrBayes ver. 3.2.7 (Ronquist et al., 2012).

For the ML analysis with 1,000 bootstrap replicates, the best fitting evolutionary models of nucleotide substitutions according to the BIC value were TIM+F+I+G4 for ITS1–2, HKY+F+G4 for *trn*L–F, K3Pu+F+G4 for *trn*G, TN+F+G4 for *rbc*L, and HKY+F+R2 for *psb*A as determined by ModelFinder (model-selection method implemented in IQ-tree) (Kalyaanamoorthy et al., 2017). Bootstrap support (BS) percentage values were calculated.

BI was performed by running two parallel analyses using the GTR+I+G model. For all datasets, the analysis consisted of four Markov chains run for five million generations, and trees were sampled every 500th generation. The first 2,500 trees in each run were discarded as burn-in; thereafter, 15,000 trees were sampled from both runs. Bayesian posterior probabilities (PPs) were calculated from the trees sampled after burn-in. The average standard deviation of split frequencies between the two runs was 0.0071 for ITS1–2, 0.0049 for *trn*L–F, 0.003 for *trn*G, 0.0047 for *rbc*L, and 0.003 for *psb*A.

## 3 Results

The infraspecific/generic and interspecific/generic variability (**Table 2**) of all five datasets were quantified as the average pairwise *p*-distances calculated in Mega XI (Tamura et al., 2021) using the pairwise deletion option for counting gaps.

**Table 2:** The value of infraspecific/generic and interspecific/generic *p*-distances for the tested nucleotide alignments.

No.	Taxon	Infraspecific and infrageneric *p*-distances, ITS/trnL/trnG/ rbcL/psbA, %	Interspecific and intergeneric *p*-distances, ITS/trnL/trnG/rbcL/psbA, %										
			1	2	3	4	5	6	7	8	9	10	11
1	*Cephaloziella* 1	2. 5/0. 9/1. 0/ 0.3/0											
2	*Cephaloziella* 2	12. 7/2. 8/n/c/ -/n/c	17. 3/3. 3/5. 9/ -/2. 5										
3	*Cephaloziella* 3	6. 9/0. 9/n/c/ n/c/n/c	20. 4/4. 0/5. 4/ 2. 3/2. 7	22. 5/4. 6/4. 5/ -/2. 1									
4	*Cephaloziella* 4	0. 7/n/c/-/ n/c/n/c	26. 7/3. 9/-/ 3. 4/2. 7	28. 1/6. 1/-/ -/4. 1	29. 2/7. 1/-/ 2. 5/2. 5								
5	*Cephaloziella* 5	-/n/c/n/c/ n/c/n/c	-/2. 8/3. 4/ 1. 0/0. 9	-/4. 7/4. 8/ -/1. 3	-/4. 9/4. 5/ 1. 8/2. 3	-/6. 9/-/ 3. 2/1. 8							
6	*Cylindrocolea*	9. 7/1. 7/1. 0/ 1. 2/0. 3	17. 9/2. 4/5. 1/ 2. 2/1. 2	20. 4/3. 7/5. 4/ -/2. 8	24. 2/4. 8/4. 7/ 2. 1/2. 3	28. 6/5. 2/-/ 3. 7/3. 4	-/4. 1/4. 2/ 1. 9/0. 9						
7	*Cephaloziopsis exigua*	n/c/0/n/c/ 0/n/c	20. 0/2. 7/4. 3/ 1. 2/1. 2	22. 5/3. 6/7. 1/ -/3. 4	27. 5/4. 2/5. 5/ 2. 9/3. 2	28. 6/5. 6/-/ 4. 1/3. 6	-/3. 0/4. 7/ 1. 7/1. 2	23. 3/3. 2/5. 9/ 3. 0/1. 7					
8	*Cephaloziella intertexta-crispata/crispata*	-/n/c/n/c/ 8. 2/1. 7	-/3. 0/4. 3/ 4. 4/1. 3	-/3. 9/7. 1/ -/3. 4	-/4. 1/5. 5/ 5. 0/2. 9	-/5. 4/-/ 6. 5/3. 7	-/3. 3/4. 7/ 4. 5/1. 1	-/3. 3/5. 9/ 4. 5/1. 3	-/0. 5/0/ 4. 3/0. 8				
9	*Kymatocalyx dominicensis*	-/n/c/-/ n/c/n/c	-/2. 0/-/ 4. 1/1. 1	-/2. 9/-/ -/2. 9	-/4. 5/-/ 3. 0/2. 1	-/4. 8/-/ 1. 5/3. 5	-/3. 6/-/ 4. 0/0. 8	-/1. 7/-/ 4. 2/0. 9	-/2. 7/-/ 4. 8/1. 6	-/3. 2/-/ 6. 6/1. 3			
10	*Cephalomitrion aterrimum*	-/-/-/ n/c/n/c	-/-/-/ 2. 1/1. 5	-/-/-/ -/3. 2	-/-/-/ 2. 0/2. 9	-/-/-/ 3. 7/3. 5	-/-/-/ 1. 6/0. 9	-/-/-/ 1. 7/1. 0	-/-/-/ 2. 9/2. 4	-/-/-/ 4. 7/1. 8	-/-/-/ 4. 5/1. 1		
11	*Cephaloziella granatensis*	-/-/-/ n/c/n/c	-/-/-/ 2. 2/2. 0	-/-/-/ -/2. 2	-/-/-/ 0. 8/2. 2	-/-/-/ 2. 2/3. 1	-/-/-/ 2. 1/1. 4	-/-/-/ 2. 3/2. 1	-/-/-/ 2. 9/2. 5	-/-/-/ 5. 5/2. 3	-/-/-/ 3. 0/2. 0	-/-/-/ 2. 1/2. 1	
12	*Obtusifolium obtusum*	0. 4/1. 0/0. 2/ 0. 6/n/c	25. 9/10. 2/15. 2/ 6. 4/4. 4	28. 0/11. 3/15. 0/ -/6. 6	32. 1/12. 0/14. 4/ 6. 2/4. 7	29. 9/11. 7/-/ 6. 0/5. 1	-/10. 7/13. 8 6. 4/3. 5	33. 3/11. 2/15. 1/ 7. 0/5. 7	27. 6/10. 3/14. 7/ 7. 3/6. 4	-/10. 1/14. 7/ 8. 6/6. 4	-/10. 6/14. 7/ 6. 6/5. 9	-/-/-/ 6. 8/6. 6	-/-/-/ 5. 9/4. 8

n/c, non-calculated value due to single specimen only; dashes, null data.

Fifty new sequences from *Cephaloziella*, *Cephaloziopsis*, *Cylindrocolea*, *Hygrobiella*, and *Obtusifolium* S.W.Arnell specimens were deposited in the NCBI GenBank (**Supplementary Table 1**), and the characteristics of the tested alignments are shown in **Table 3**. The log-likelihood values for the ML analysis and Bayesian analysis of all datasets are presented in **Table 4**.

**Table 3:** The characteristics of the tested nucleotide sequence alignments.

Locus	Total sites	Conservative sites		Variable sites		Parsimony-informative sites	
		Base pairs	%	Base pairs	%	Base pairs	%
ITS1–2	1,115	373	33.45	702	62.96	550	49.33
*trn*L–F	510	285	55.88	214	41.96	160	31.37
*trn*G	735	417	56.73	282	38.37	260	35.37
*rbc*L	1,379	1,041	75.49	336	24.37	217	15.74
*psb*A	1,420	1,097	77.25	308	21.69	219	15.42

**Table 4:** The log-likelihood in the ML analysis and in Bayesian analysis (arithmetic mean) of the tested nucleotide sequence alignments.

Locus	ML analysis	Bayesian analysis	
		Run 1	Run 2
ITS1–2	−10,148.03	−10,192.85	−10,193.46
*trn*L–F	−3,305.70	−3,402.95	−3,401.41
*trn*G	−3,280.16	−3,307.68	−3,308.73
*rbc*L	−5,473.61	−5,511.14	−5,506.99
*psb*A	−5,042.57	−5,074.57	−5,074.72

ML, maximum likelihood.

**Figure 1:**
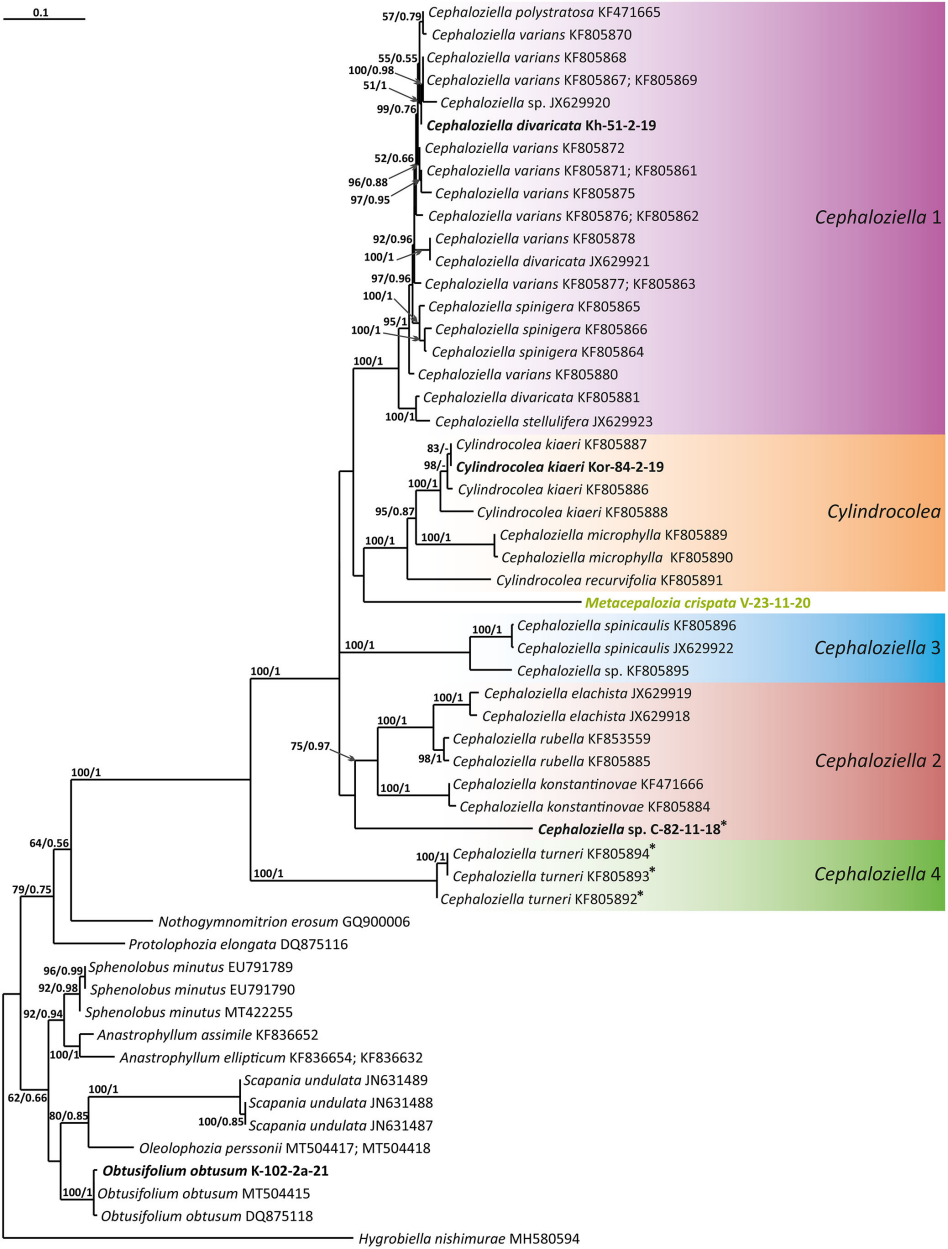
Phylogram obtained from a Bayesian analysis of the listed taxa based on the ITS1–2 dataset. The values of bootstrap support from the ML analysis and Bayesian posterior probabilities greater than 50%/0.50 are indicated. Taxon names and GenBank accession numbers or vouchers (for the samples studied by the authors) are provided. Asterisks mark taxa with different positions on ML tree. The bold font indicates the specimens sequenced for the present paper. ML, maximum likelihood.

**Figure 2:**
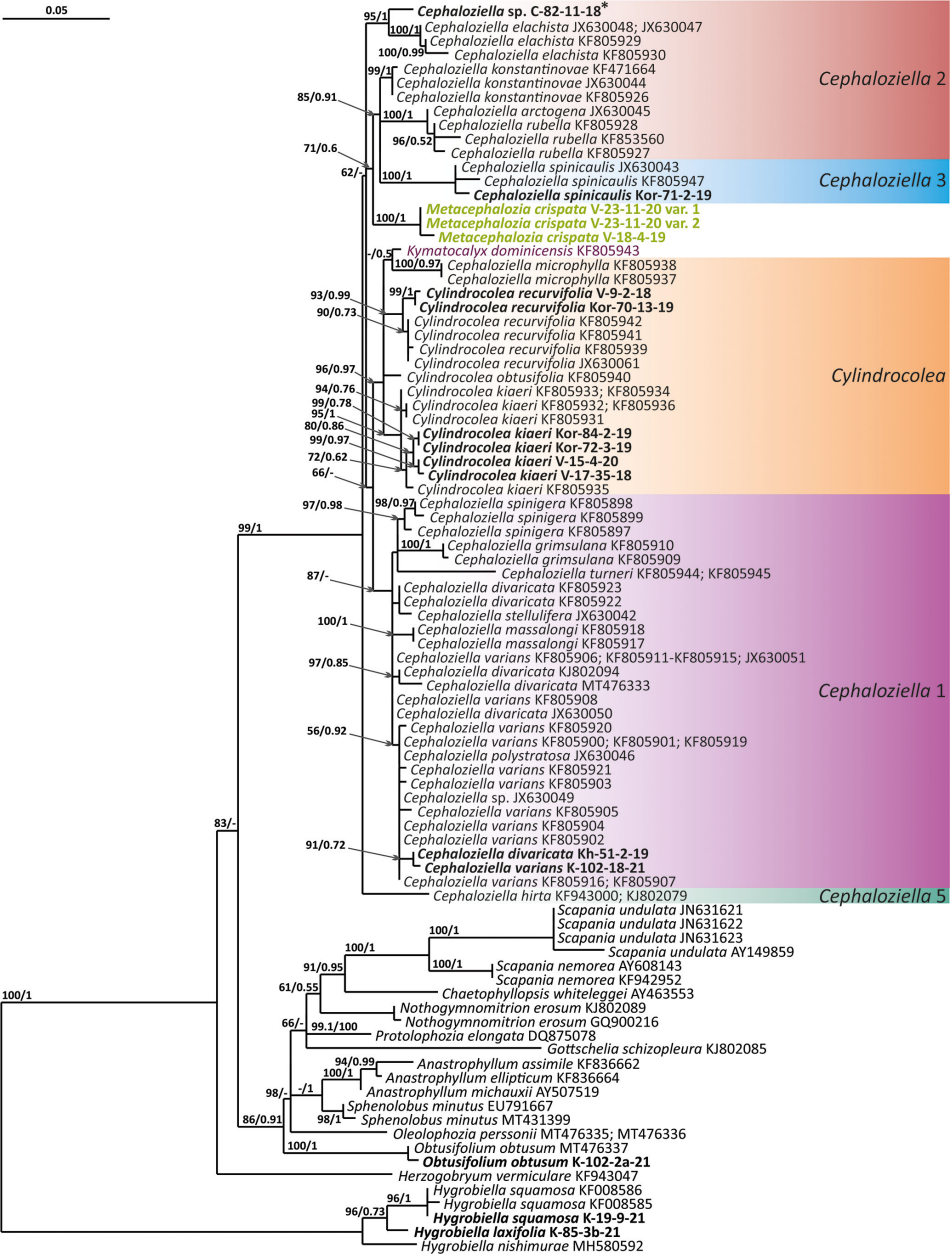
Phylogram obtained from a Bayesian analysis for the listed taxa based on the *trn*L-F dataset. The values of bootstrap support from the ML analysis and Bayesian posterior probabilities greater than 50%/0.50 are indicated. Taxon names and GenBank accession numbers or vouchers (for the samples studied by the authors) are provided. Asterisks mark taxa with different positions on ML tree. The bold font indicates the specimens sequenced for the present paper. ML, maximum likelihood.

**Figure 3:**
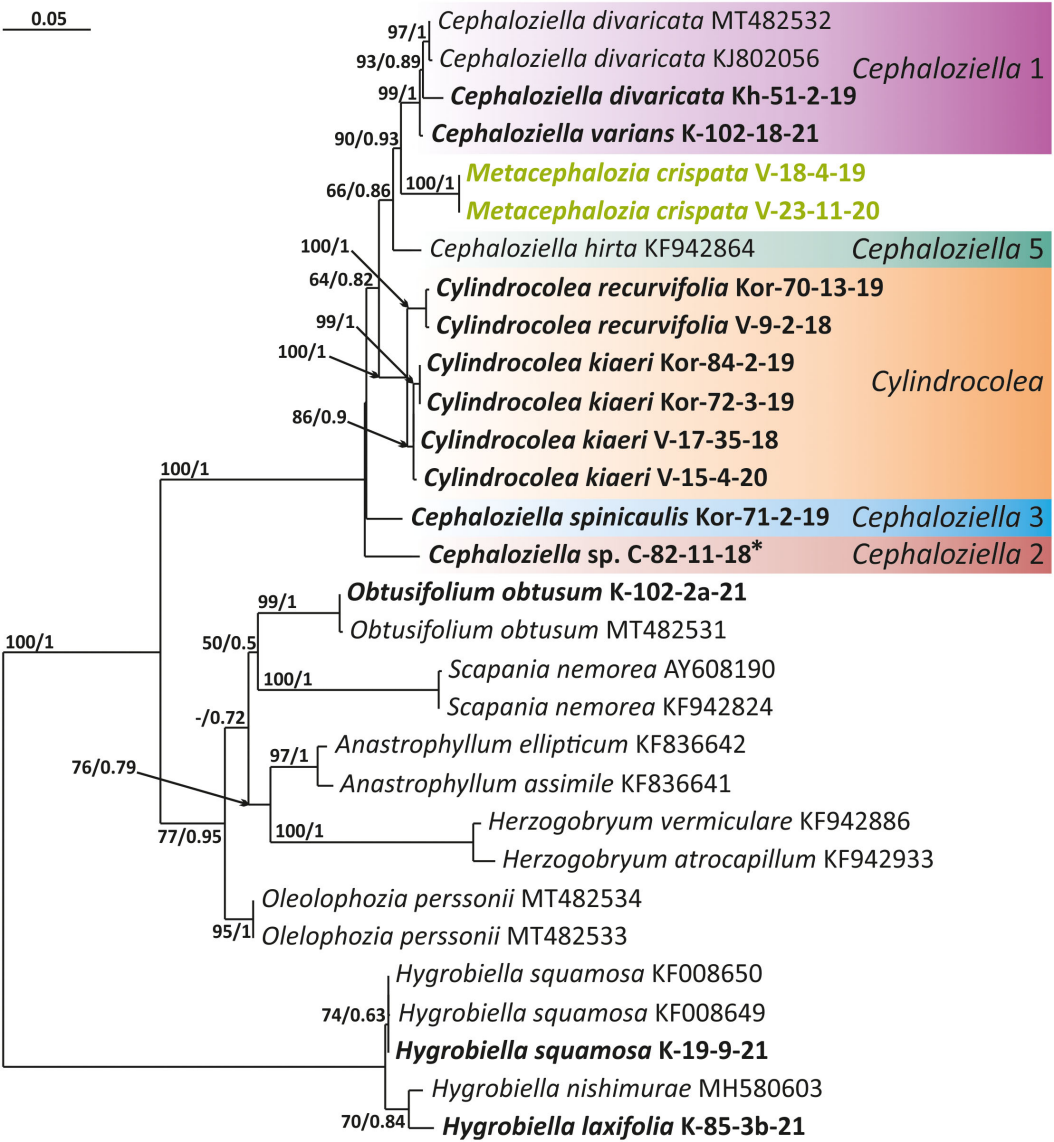
Phylogram obtained from a Bayesian analysis for the listed taxa based on the *trn*G-intron dataset. The values of bootstrap support from the ML analysis and Bayesian posterior probabilities greater than 50%/0.50 are indicated. Taxon names and GenBank accession numbers or vouchers (for the samples studied by the authors) are provided. Asterisks mark taxa with different positions on ML tree. The bold font indicates the specimens sequenced for the present paper. ML, maximum likelihood.

**Figure 4:**
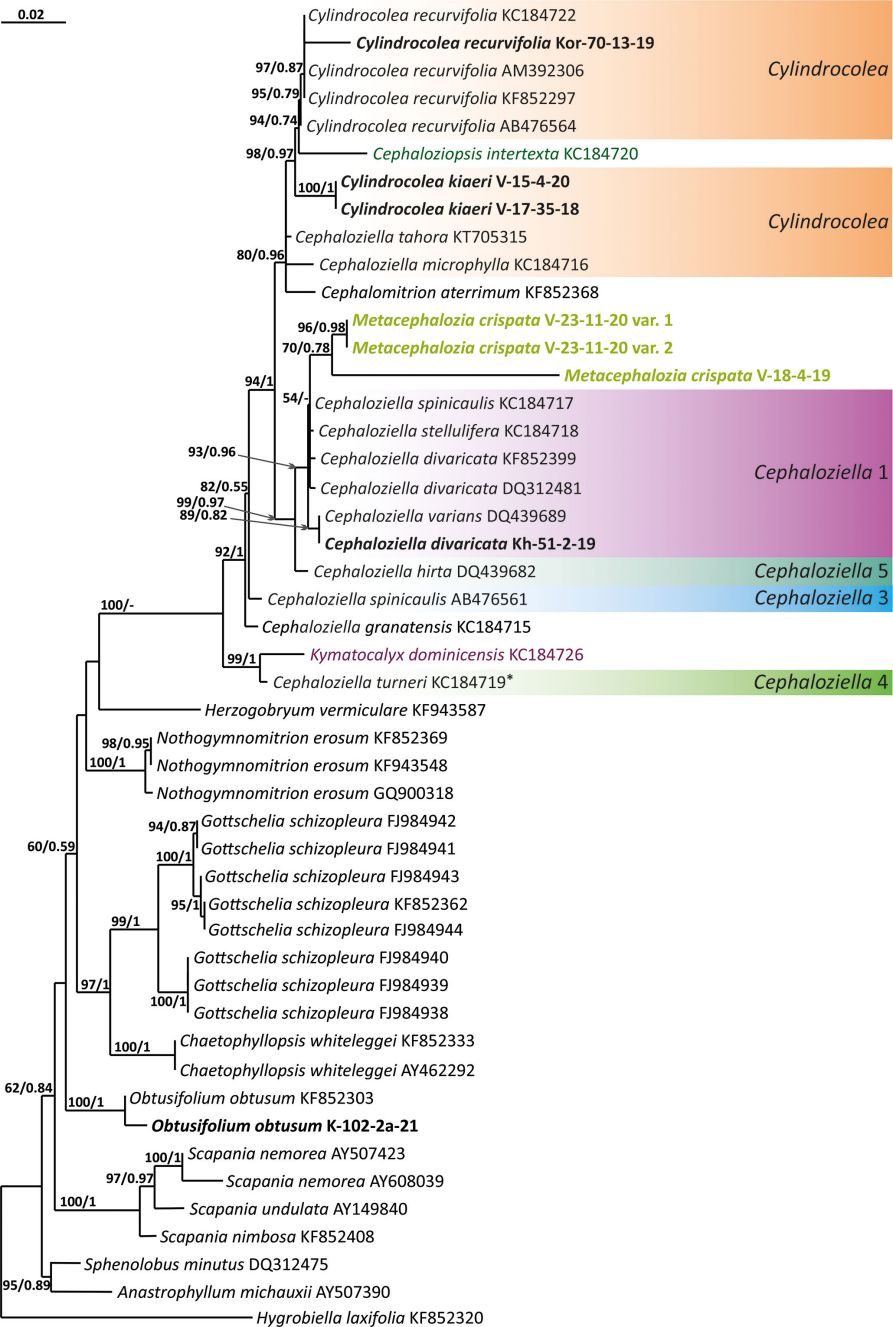
Phylogram obtained from a Bayesian analysis for the listed taxa based on the *rbc*L dataset. The values of bootstrap support from the ML analysis and Bayesian posterior probabilities greater than 50%/0.50 are indicated. Taxon names and GenBank accession numbers or vouchers (for the samples studied by the authors) are provided. Asterisks mark taxa with different positions on ML tree. The bold font indicates the specimens sequenced for the present paper. ML, maximum likelihood.

**Figure 5:**
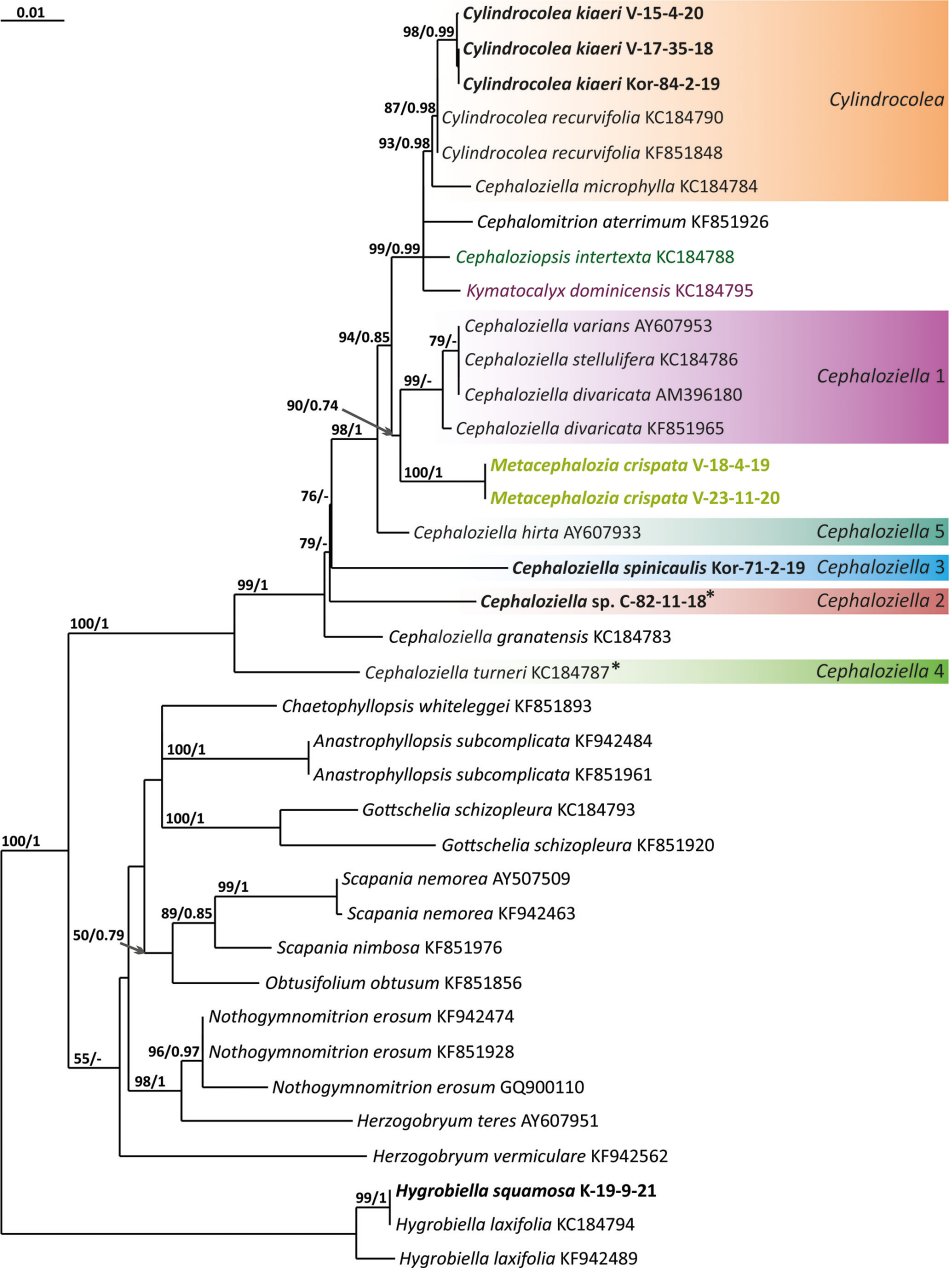
Phylogram obtained from a Bayesian analysis for the listed taxa based on the *psb*A dataset. The values of bootstrap support from the ML analysis and Bayesian posterior probabilities greater than 50%/0.50 are indicated. Taxon names and GenBank accession numbers or vouchers (for the samples studied by the authors) are provided. Asterisks mark taxa with different positions on ML tree. The bold font indicates the specimens sequenced for the present paper. ML, maximum likelihood.

The tree topologies obtained by the two methods were slightly different, so all of them are presented here. **Figures 1**–**5** show the phylogenetic trees based on the ITS1–2, *trn*L–F, *trn*G, *rbc*L, and *psb*A datasets retained under ML analysis, along with BS values from the ML analysis and the Bayesian PPs. The differences concern the variable position of some branches of the trees constructed by the two methods. Despite small inconsistencies, the following clades are clearly distinguished on all trees: *Cephaloziella* 1 (ITS1–2, *trn*L–F, *trn*G, *rbc*L, and *psb*A), *Cephaloziella* 2 (ITS1–2, *trn*L–F, *trn*G, and *psb*A), *Cephaloziella* 3 (ITS1–2, *trn*L–F, *trn*G, *rbc*L, and *psb*A), *Cephaloziella* 4 (ITS1–2, *rbc*L, and *psb*A), *Cephaloziella* 5 (*trn*L–F, *trn*G, *rbc*L, and *psb*A), and *Cylindrocolea* (ITS1–2, *trn*L–F, *trn*G, *rbc*L, and *psb*A). Detailed information on these clades is provided in the Discussion section.

## 4 Discussion

The first to demonstrate a sister relationship between Cephaloziellaceae and the Scapaniaceae–Lophoziaceae complex based on two chloroplast genes was De Roo et al. (2007), showing that *Cephaloziella* is more closely related to *Lophozia* (Dumort.) Dumort. and *Scapania* (Dumort.) Dumort. and then to the bulk of taxa later described as Anastrophyllaceae (Söderström et al., 2010). Although Anastrophyllaceae nevertheless seems to be more closely related to Scapaniaceae and Lophoziaceae than Cephaloziellaceae to the last two families, the problem was raised. In the most general approximation, the Cephaloziellaceae phylogeny was reviewed by Vilnet et al. (2012) based on a comparison of the nucleotide sequences of ITS1–2 nuclear and *trn*L–F chloroplast DNA. The authors emphasized the instability of the position of the family Cephaloziellaceae in trees constructed by different methods. This study is remarkable due to the obtained evidence that the family Cephaloziellaceae is molecularly distinct from the complex of other families and genera with which it was associated in later papers by other authors. Mamontov and Vilnet (2017) described a new species (*Cephaloziella konstantinovae* Mamontov et Vilnet) and briefly reviewed the Cephaloziellaceae system on the basis of reconstruction from the same two genes, ITS1–2 and *trn*L–F, using newly obtained materials. Topologies obtained for *Cephaloziella* s.l. by different methods (Bayesian analysis and maximum parsimony) differ in details, but they clearly show that the genus *Cylindrocolea* should also be included in *Cephaloziella* s.l. since it is nested within it. However, the authors did not make any taxonomic decisions in light of the data obtained, except for the recommendation to include *Cylindrocolea recurvifolia* (Steph.) Inoue within the genus *Cephaloziella* and treat it as *Cephaloziella recurvifolia* (Steph.) S. Hatt.

The paper by Feldberg et al. (2013), devoted to the estimation of divergence times in liverworts and based on the *rbc*L and *psb*A genes, was based on a dataset of Cephaloziellaceae smaller than those in the papers by Vilnet et al. (2012) and Mamontov and Vilnet (2017) cited above. The following clades are distinguished within Cephaloziellaceae: 1) *C. recurvifolia*, *Cephaloziopsis intertexta* (Gottsche) R.M.Schust., and *Cephaloziella microphylla* (Steph.) Douin; 2) *Cephaloziella stellulifera* (Taylor ex Carrington et Pearson) Croz., *Cephaloziella divaricata* (Sm.) Schiffn., and *Cephaloziella spinicaulis* Douin; 3) *Cephaloziella granatensis* (J.B.Jack ex Steph.) Fulford; and 4) *Kymatocalyx dominicensis* (Spruce) Váňa and *Cephaloziella turneri* (Hook.) Müll.Frib. Given that the datasets in the works of Vilnet et al. (2012) and Mamontov and Vilnet (2017), on the one hand, and Feldberg et al. (2013), on the other, are practically not based on the same specimens and provide different topologies, the problems of the Cephaloziellaceae system actually remain unresolved.

Therefore, it was an ambitious goal to seek a broad interpretation of the Cephaloziellaceae in the paper by Váňa et al. (2013), citing the work by Feldberg et al. (2013) as the basis for their solutions, albeit with a remark (Váňa et al., 2013: 1): “The only molecular study dedicated to this group including a fair number of taxa is Feldberg et al. (2013), but as it is a study of divergence time and does not include confidence values, it is difficult to draw too many conclusions from it.” Meanwhile, the authors rightly noted that “As circumscribed here, Cephaloziellaceae is morphologically heterogeneous and is probably best treated as a ‘superfamily’. Morphologically it is difficult to defend such a diverse family and it should probably be separated into further families.” In the list given by Váňa et al. (2013), the genera are conditionally divided into two groups: those whose position is unclear and which belong to Cephaloziellaceae in the broad sense of the interpretation of this family and those that belong to the Cephaloziellaceae “core” group. The last group included eight genera: *Amphicephalozia*, *Cephalojonesia* Grolle, *Cephaloziella*, *Cephalomitrion*, *Cephaloziopsis*, *Cylindrocolea*, *Gymnocoleopsis*, and *Kymatocalyx* Herzog. The group belonging to the Cephaloziellaceae in a broad sense includes only 10 genera (as in the cited paper): *Allisoniella* E.A.Hodgs., *Anastrophyllopsis* (R.M.Schust.) Váňa et L.Söderstr., *Chaetophyllopsis* R.M.Schust., *Gottschelia* Grolle, *Herzogobryum* Grolle, *Lophonardia* R.M.Schust., *Nothogymnomitrion* R.M.Schust., *Obtusifolium* S.W. Arnell, *Oleolophozia* L.Söderstr., De Roo et Hedd., and *Protolophozia* (R.M.Schust.) Schljakov. It is worth mentioning that these two groups are strikingly different in appearance. The Cephaloziellaceae “core” group includes very small plants, with distanced leaves and a seta structure (where known) of 4 + 4, 4 + 8, or similar (corresponding to the reported “archetype” of *Cephaloziella* of 4 + 4), while another group includes plants much larger and rather “lophozioid” in appearance, with massive setae. This is also confirmed by the fact that genera such as *Obtusifolium*, *Oleolophozia*, *Protolophozia*, and *Lophonardia* were earlier (in the era of “morphological taxonomy”) commonly referred to *Lophozia* in a broad interpretation of that genus.

A robust step forward in understanding the phylogenetic relationships of the Cephaloziellaceae was made by Patzak et al. (2016). The authors, based on the analysis of an *rps*4–*rbc*L dataset, showed the synonymy of Chonecoleaceae R.M.Schust. ex Grolle and Cephaloziellaceae, although the morphological structure of the seta was not quite typical (eight epidermal and four inner rows, as in *Cephalomitrion*) for Cephaloziellaceae. However, the Cephaloziellaceae “core” group actually shows deviations and diversity in morphology, even in the already-known species. Patzak et al. (2016) indicated that the structure of the seta within the family (in a narrow sense) varies considerably, namely, (l.c.: 98): “4, rarely (6–)8(–9) epidermal and (3–)4(–14) inner rows”. In addition to clarifying the taxonomic “amount” of the Cephaloziellaceae, based on the same *rps*4–*rbc*L dataset, the authors came to the conclusion that the Scapaniaceae family should be interpreted much more widely than is commonly accepted even in broad interpretations of the family (when Lophoziaceae, Anastrophyllaceae, and Scapaniaceae are combined together). The authors moved into the latter group *Herzogobryum*, *Nothogymnomitrion*, *Anastrophyllopsis*, *Chaetophyllopsis*, *Gottschelia*, and *Obtusifolium*, which, 3 years earlier in the concept by Váňa et al. (2013), were considered members of the Cephaloziellaceae superfamily, with the note that (l.c.: 1) “Morphologically it is difficult to defend such a diverse family and it should probably be separated into further families”. Thus, instead, the Cephaloziellaceae superfamily was revised to include Scapaniaceae s.l. (also a superfamily), thus including heterogeneous elements.

In addition to those above, another question remains unresolved: if the topologies obtained by Vilnet et al. (2012) and Mamontov and Vilnet (2017), based on ITS1–2 and *trn*L–F analyses, differ from the topologies obtained from the *rps*4–*rbc*L dataset, can one of these points of view be considered more correct than the other if no cross-comparisons were conducted?

Later, while studying the molecular genetic relationships of *Konstantinovia* Bakalin et Fedosov (a hitherto monotypic genus) described as new to science, Bakalin et al. (2021a) found it to be most closely related to the genus *Obtusifolium*, prompting a new attempt to determine the taxonomic position of these two genera. This task was solved on the basis of a comparison of four genes: the nuclear ITS region and the chloroplast *rps*4 gene, *trn*L–F region, and *trn*G-intron. As a result, it was shown that 1) Anastrophyllaceae is clearly distinguished in the system Scapaniaceae s.l. plus Cephaloziellaceae s.l.; 2) Lophoziaceae and Scapaniaceae differ less prominently (actually, Lophoziaceae is nested within Scapaniaceae; however, under certain conditions, these families can be treated separately); 3) *Obtusifolium* plus *Konstantinovia* should be segregated into a new family, which is much more closely related to the Scapaniaceae plus Anastrophyllaceae, but not to the Cephaloziellaceae; and 4) *Oleolophozia* should probably also be placed in a separate family (taxonomic decisions on the last matter have not been made).

Our present study began with the need to determine the position of plants collected in Vietnam, which we identified as *C. exigua* (Inoue) R.M. Schust. et Inoue, a name that does not appear in the current literature; these plants are commonly known as *Cephaloziella crispata* N.Kitag. The history of the interpretation of this taxon is rather confusing. It has been described as *Metacephalozia exigua* Inoue (Inoue, 1973). In the original author’s interpretation, this species (and genus) occupies an intermediate position in terms of morphology between Cephaloziaceae and Cephaloziellaceae (Inoue, 1973). A year later, Inoue and Schuster (1974) published a paper in which, based on a comparison of the morphology of a little-known Neotropical *Cephaloziopsis* with *Metacephalozia* Inoue, they came to the conclusion that the latter deserved only the rank of a subgenus within the former (as an older name). The result was also a new combination: *C. exigua*. At the same time, the authors of the cited work emphasized a serious difference in the distribution of the two subgenera (Neotropics versus warm temperate East Asia), associated with minor morphological differences.

Later, Katagiri and Furuki (2012) stated (in one sentence) that *C. crispata*, described by Kitagawa in 1969, is synonymous with *M. exigua*, described by Inoue in 1973. As a result of formal synonymization, this species was placed in the Japanese liverwort checklist under the name *C. crispata*. The authors (Katagiri and Furuki, 2012) did not express their opinion regarding the status of *Metacephalozia* or *Cephaloziopsis*. Interestingly, given the similar morphologies of *Cephaloziopsis* s. str. and *Metacephalozia* (which authors of the Japanese checklist did not contest), it would be logical to assume that if one can be declared a synonym of *Cephaloziella* without much doubt, then it would be better to synonymize the *Cephaloziopsis* genus with *Cephaloziella* as well. However, the genus (or subgenus) *Metacephalozia* has simply disappeared, while *Cephaloziopsis* “continued to exist”. *Cephaloziopsis* subg. *Cephaloziopsis*, as recognized in the World Checklist (Söderström et al., 2016), including one species, *C. intertexta*. The second species of the genus [following the interpretation by Schuster (1972)] *Cephaloziopsis schistophila* (Spruce) R.M. Schust., as foreseen by Schuster (l.c.: 182), “I have had one of the two as a subspecies of *C. schistophila*, and, indeed, subspecific segregation may prove more nearly correct”, was eventually synonymized with the earlier *C. intertexta*, and the latter name appears on the World Liverwort Checklist (Söderström et al., 2016).

In light of our data on phylogenetic relationships between different groups within Cephaloziellaceae s. str., two options are possible: 1) to consider all genera for which there are nucleotide sequences forming a single unit (including all previously segregated genera, e.g., *Cephaloziopsis* and *Cylindrocolea*) as the genus *Cephaloziella* s.l. and 2) along with the revisiting of existing genera, it is necessary to segregate new genera. Taking into account the general trend and genetic distance values (**Table 2**) between the identified clades, we chose the second option. Below, we describe clades on phylogenetic trees as harbingers for the possible taxonomic solutions within the Cephaloziellaceae. The morphological background of the obtained clades remains not entirely clear. Probably leaf attachment (oblique or almost transverse) is an important character in the taxonomy of Cephaloziellaceae. Some aberrations in the structure of the sporophyte are also confirmed as significant characters in the taxonomy of Cephaloziellaceae, but there are still very little data on this issue. It seems to us that the most significant criteria are not even morphological, but geographical ones, namely, patterns of distribution, since the distinguished clades (in the most general terms) may differ in the distribution of the species included in them, for example, South American, Paleotropical, mainly East Asian, and North Holarctic. This issue deserves further detailed study.

Clade *Cephaloziella* 1 is the genus *Cephaloziella* in the narrow sense. This clade contains the type species of the genus (*C. divaricata*). In most trees, this clade is quite well isolated from others, and its composition is stable: *C. divaricata*, *Cephaloziella varians* (Gottsche) Steph., *Cephaloziella spinigera* (Lindb.) Jørg., *Cephaloziella polystratosa* (R.M.Schust. et Damsh.) Konstant., *Cephaloziella grimsulana* (J.B.Jack ex Gottsche et Rabenh.) Lacout., *C. stellulifera*, and *Cephaloziella massalongi* (Spruce) Müll.Frib. In general, this group seems to correspond with the subgenus *Cephaloziella* of the genus if a broad interpretation is accepted.

*Cylindrocolea* described by Schuster (Schuster, 1969: 666) is treated on the World Checklist as housing 16 species (Söderström et al., 2016). Only a few of these genera were tested by molecular genetic methods; the type of the genus (*Cylindrocolea chevalieri* (Steph.) R.M. Schust.) was also not tested. In general terms, the genus differs from *Cephaloziella* s str. (cf. Schuster, 1972) as follows: 1) very obliquely inserted leaves, 2) leaf-free antical sector of the stem, 3) absence of gemmae, and 4) wide or only weakly contracted perianth mouth. Taking into account the available data on the molecular relationships of species, *C. recurvifolia* can be reliably assigned to this genus. *Cylindrocolea kiaeri* (Austin) Váňa also belongs to this genus. Moreover, it should be noted that although we call the sequenced specimens *C. kiaeri*, all sequenced materials (both from us and available from GenBank) originate from East Asia, Indochina, and Malaysia. Meanwhile, the species has been described from Africa. The identity of the Asian and African materials may be questioned, although at the moment we do not have any confirmation of this point of view (a brief discussion on it can be found in Bakalin et al. (2023). The same group clearly includes *C. microphylla*, previously placed in *Cephaloziella* subg. *Prionolobus* (Spruce) Müll. Frib. (type species is *C. turneri*). *C. microphylla* clearly differs from other representatives of *Cylindrocolea*, mainly in the abundance of mammillose protrusions and outgrowths on the abaxial leaf surface. Despite outwardly significant differences, molecular genetic data for four genes (ITS, *trn*L–F, *rbc*L, and *psb*A) agree on the need to refer this species to *Cylindrocolea*.

There is much less reason to recognize *Kymatocalyx* as a distinct genus. Four species are known in the genus (type species *Kymatocalyx stolonifer* Herzog = *K. dominicensis*). Molecular data are available for the type species only for *trn*L–F (for which the species is unequivocally included in the genus *Cylindrocolea*) and *rbc*L (where *Kymatocalyx* is found to be related to *C. turneri*). Given the incongruence found, we refrain from making taxonomic decisions on this matter. However, we note that the phrase of Schuster (Schuster, 1972: 177) “The genus *Kymatocalyx* is at once distinct from other taxa referred to the Cephaloziellaceae in having quite unlobed leaves and female bracts” can only be taken into account with reservations. For example, *C. recurvifolia* often has very shallowly emarginate leaves. The same can be said of *C. chevalieri*. Therefore, if we compare the variability of the leaf shape in *Cylindrocolea* in the series from *C. kiaeri* to *C. recurvifolia*, we can assume that the variants of *Kymatocalyx* are not sufficiently unique for morphological substantiation of its interpretation as a separate genus. However, due to data limitations, we refrain from formally synonymizing *Kymatocalyx* with *Cylindrocolea.*

The *Cephaloziella* 2 group in the ITS-based phylogenetic tree includes *Cephaloziella elachista* (J.B.Jack ex Gottsche et Rabenh.) Schiffn., *C. konstantinovae*, and *Cephaloziella rubella* (Nees) Warnst. The same species also formed a clade in the ITS1–2 + *trn*L–F trees in the study by Mamontov and Vilnet (2017). In **Figure 1**, this group is adjacent with low support to a potentially new species designated *Cephaloziella* sp. (C-82-11-18). The *trn*L–F-based tree (**Figure 2**) shows *C. rubella*, *Cephaloziella arctogena* (R.M.Schust.) Konstant., and *C. konstantinovae* with *C. spinicaulis* in a clade (BS *=* 85, PP *=* 0.91) sister to *C. exigua*, while *C. elachista* and *C.* sp. (C-82-11-18) form a sister branch to all Cephaloziellaceae s. str., except *Cephaloziella hirta* (Steph.) R.M.Schust.

*Cephaloziella* sp. (C-82-11-18) forms a sister branch to all Cephaloziellaceae included in the analysis in the *trn*G-based phylogenetic tree. The sub-basal position is typical for the same species in the *psb*A-based tree. It is obvious that the complex *C. konstantinovae*–*C. rubella*–*C. arctogena* deserves to be allocated to an independent genus; however, at present, we refrain from nomenclatural combinations due to 1) the ambiguity of phylogenetic relationships with *C. elachista* and 2) the ambiguity of morphological criteria delimiting the group (this is mainly due to the unstable position of *C. elachista*).

*C. spinicaulis* occupies a quite isolated position in the ITS, *trn*G, *rbc*L, and *psb*A trees. This is inconsistent with the morphological expectations of some scholars. Schuster (1980) placed this species in the section *Bissaceae* R.M. Schust. (=sect. *Cephaloziella* s. str.), together with the type species of the genus *C. divaricata*. Douin (1920) had conducted the same even before, placing this species in the “Groupe du *C. starkii*” (=*C. divaricata*). However, Schuster (l.c.) indicates that this species has a distanced position in the section due to its rigid texture, rather small leaves relative to the stem diameter, and a main stem having numerous spines that are 1–4 cells long. In addition, Schuster (Schuster, 1980: 107) wrote, “*C. spinicaulis* appears to be only remotely allied to the sub-Antarctic and Antarctic *C. hispidissima* R.M. Schust.” (=*Cephaloziella verrucosa* Steph., cf. Váňa et al., 2014). In the World Checklist (Söderström et al., 2016), the species is placed in the subgenus *Cephaloziella*, which, however, cannot be accepted according to the available topologies. This species, given the adoption of the generic status for *Kymatocalyx* and *Cylindrocolea*, must be allocated to a separate genus. At the same time, the relationship with *C. verrucosa* needs to be studied, and thus far, we have refrained from formally transferring the latter from one genus to another. We propose naming the new genus that we have identified *Douiniella*, in honor of Charles Isidore Douin (1858–1944), an outstanding expert on *Cephaloziella* in the first third of the 20th century. The genus is hitherto monotypic.

*Douiniella* Bakalin, Maltseva, Troitzk. gen. nov.

Description. Plants dioicous, of rigid texture, with dense and numerous spines covering the stem and adaxial leaf surface.

Type species: *Douiniella spinicaulis* Bakalin, Maltseva, Troitzk. *comb nov.* Basionym: *C. spinicaulis* Douin, Mém. Soc. Bot. France 29: 62, 1920 (Douin, 1920)

In the ITS, *rbc*L, and *psb*A trees (**Figures 1**, **4**, **5**), *C. turneri* occupies a sister position to all other *Cephaloziella* and should also be considered a separate genus. Since the type species for the genus *Prionolobus* is *Prionolobus turneri* (Hook.) Schiffn. (cf. Grolle, 1976), it is reasonable to maintain the generic status for the taxon appearing in Söderström et al. (2016) as *Cephaloziella* subg. *Prionolobus*. At the same time, however, it must be taken into account that of the eight species referred to this subgenus in the World Checklist (Söderström et al., 2016), at least some definitely cannot be assigned. For example, *C. microphylla* reliably belongs to *Cylindrocolea* following the obtained topologies. In addition, in the two available trees, those for *rbc*L and *psb*A, *C. granatensis* does not form a common clade with *C. turneri*; instead, with the exception of the latter and *Kymatocalyx* (in the *rbc*L tree), it is a sister to all other *Cephaloziella*. No molecular data are available for the remaining *Prionolobus* species. Therefore, *Prionolobus* reliably forms a genus, but its content (except for the type species) remains unclear.

*Cephaloziella* subg. *Evansia* (Douin et Schiffn.) Müll.Frib., with the type species *Cephaloziella dentata* (Raddi) Steph., includes (according to the list of Söderström et al. (2016) *C. hirta*. Apparently, this subgenus should also be treated as a separate genus (that should be supplied with the new generic name since *Evansia* is the later homonym of the previous *Evansia* Salisb. ex Decne); however, since we do not have data on the phylogenetic relationships of *C. dentata*, we refrain from making taxonomic decisions on this matter. However, it is worth mentioning that the morphologies of the two taxa (*C. dentata* and *C. hirta*) are very similar. The main difference between *C. dentata* and *C. hirta* is in the inflorescence: plants are dioicous in *C. dentata* but autoicous in *C. hirta*. In addition, *C. dentata* is mostly European, but with some reports from China (Hunan, Jiangxi Provinces), Japan, Tasmania, British Columbia, and Mexico (available from https://www.catalogueoflife.org/data/taxon/5XK7F), while *C. hirta* is Australasian, also with some reports from New Zealand North Island and Juan Fernández Island (available from (https://www.catalogueoflife.org/data/taxon/5XK7G).

Finally, the problem that initiated this work in general terms should be discussed. It was unclear how, in light of the currently accepted treatment (Söderström et al., 2016), to interpret the independence of *Cephaloziopsis* and *Metacephalozia*. On the one hand, the authors of the World Checklist (Söderström et al., 2016) agreed that *Metacephalozia* is a synonym of the genus *Cephaloziella* and not the same genus but the same named (type) subgenus. On the other hand, no one synonymized monotypic *Cephaloziopsis* with *Cephaloziella*. Third, no one has disputed that *Metacephalozia* and *Cephaloziopsis* are not the same genus. Thus, a contradiction arose, which we tried to resolve by molecular methods.

**Figure 6:**
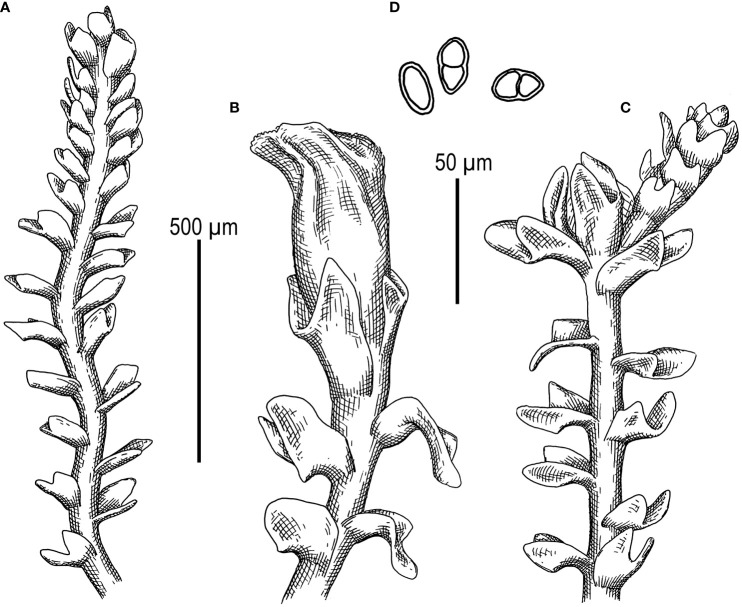
*Metacephalozia crispata* (N.Kitag.) Bakalin, Maltseva, Troitzk. **(A)** Shoot, fragment, dorsal view. **(B)** Perianthous shoot, fragment, dorsal view. **(C)** Shoot with lateral branch, fragment, dorsal view. **(D)** Gemmae. Scales: 500 µm for panels **(A–C)** and 50 µm for **(D)** All from V-23-11-20 (VBGI).

**Figure 7:**
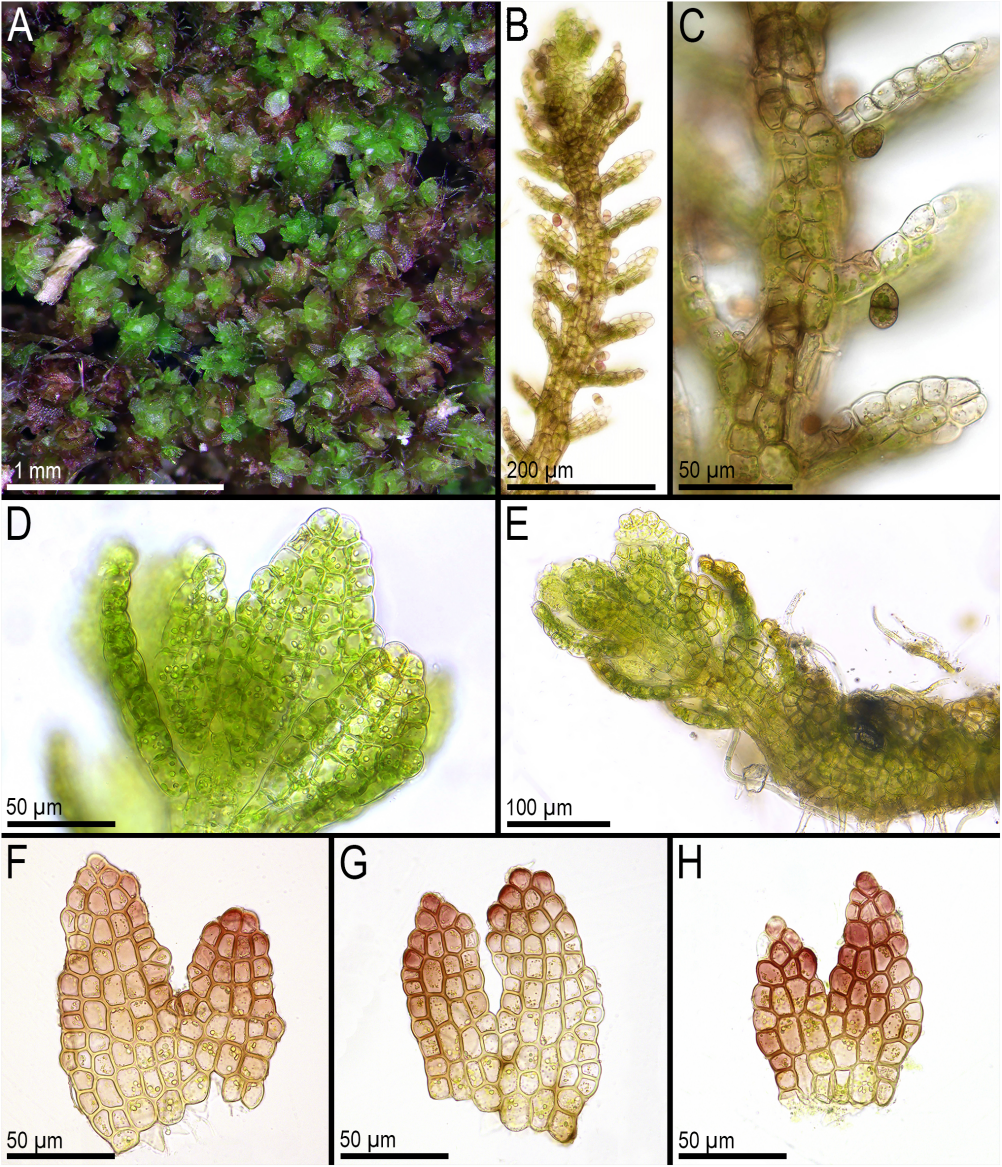
*Metacephalozia crispata* (N.Kitag.) Bakalin, Maltseva, Troitzk. **(A)** Mat. **(B, C)** Gemmiparous shoot, fragment, ventral view. **(D)** Oil bodies in leaf cells near shoot apex. **(E)** Plagiotropic shoot, fragment. **(F–H)** Leaves with oil bodies (mostly destructed). **(A, F–H)** From V-18-4-19. **(C, B)** From V-23-11-20. **(D, E)** From V-23-21-22. All from VBGI.

**Figure 8:**
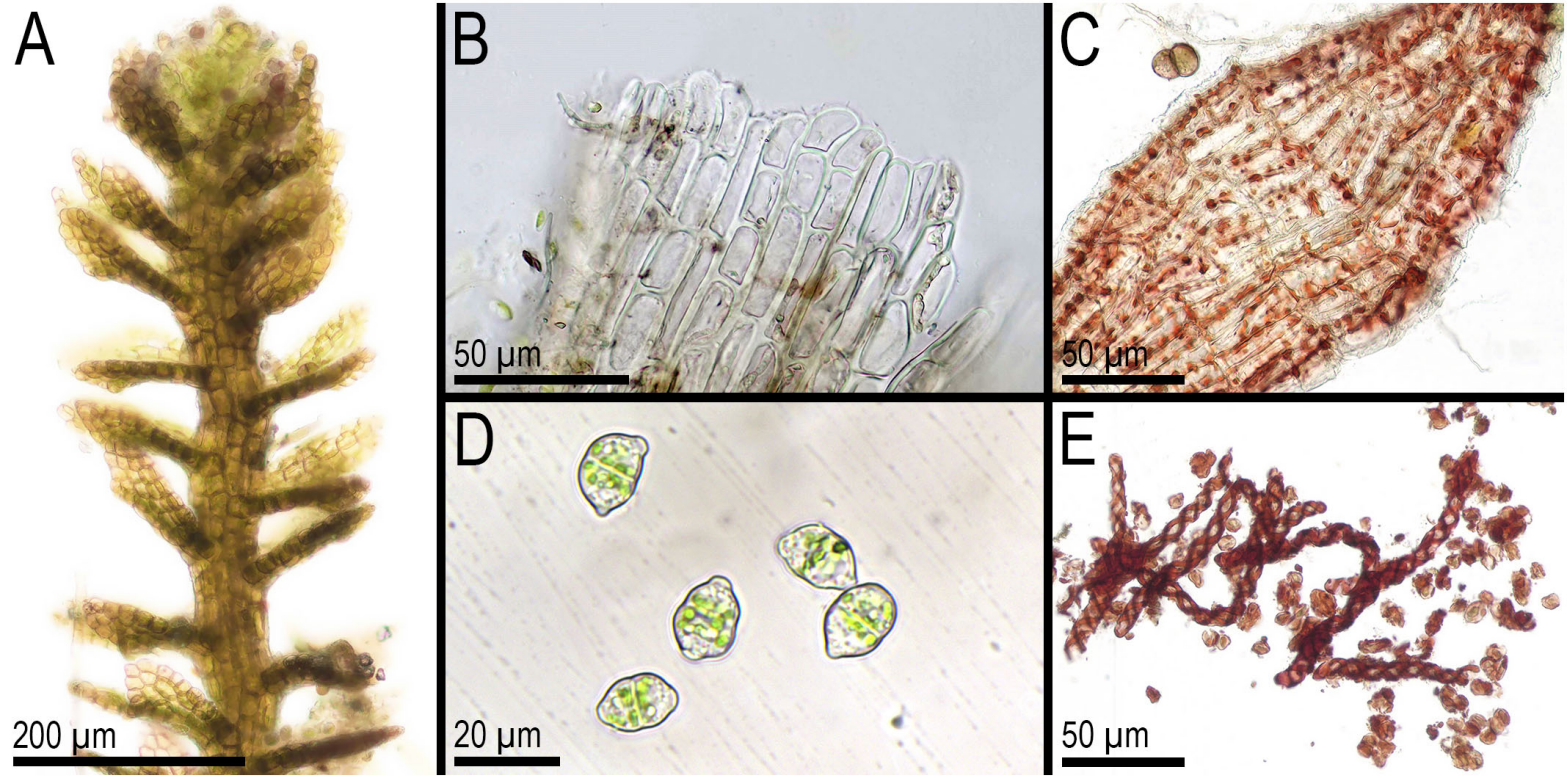
*Metacephalozia crispata* (N.Kitag.) Bakalin, Maltseva, Troitzk. **(A)** Shoot, fragment, dorsal view. **(B)** Perianth mouth. **(C)** Outer cells of capsule lobe. **(D)** Gemmae. **(E)** Spores and elaters (spores are distinctly collapsed). **(A–C, E)** From V-23-11-20. **(D)** From V-6-20-22. All from VBGI.

First, *C. crispata* and *M. exigua* are undoubtedly conspecifics. The first is (according to the type of materials) simply a form of the second arising in shadier habitats. Our studies show that this species is very polymorphic and quite common in the mountains of North Vietnam, where it grows on moist, bare loamy soil (usually without shading or under weak shading). In other words, it tends to prefer habitats favored by many members of *Cephaloziella* s. str. in the global North. Ecologically determined modifications are genetically almost identical, as the constructed topologies show. The position of *M. exigua* (=*C. crispata*) varies from tree to tree. According to the ITS data (**Figure 1**), the species forms a sister branch to all *Cylindrocolea*. According to the *trn*L–F tree (**Figure 2**), this species is sister to the group *C. arctogena*-*konstantinovae* (*Cephaloziella* 2) + *C. spinicaulis* (*Douiniella*, which we propose). According to the *trn*G, *rbc*L, and *psb*A trees (**Figures 3**–**5**), this species is sister to *Cephaloziella* s. str. (*C. divaricata* and *C. varians*) (*Cephaloziella* 1 clade).The position of *M. exigua* described above differs greatly from the position of *C. intertexta* (the second species of *Cephaloziopsis* s.l.), which, according to *rbc*L, is sister to *Cylindrocolea* or close to it in the *rbc*L-based reconstruction. Thus, if the genus *Cylindrocolea* is distinguished from *Cephaloziella* s. str. (and even more so if other genera within Cephaloziellaceae s. str. are recognized), then it should be accepted that *C. intertexta* and *M. exigua* belong to different genera. Moreover, given the variable position of both genera in phylogenetic trees based on different genes, neither of them can be confidently combined with either the genus *Cephaloziella* (as it is accepted here, i.e., in the narrow sense) or the genus *Cylindrocolea*, and at the moment, the best option would be to recognize the independence of the two. At the same time, since *C. crispata* was described earlier than *M. exigua*, it is necessary to propose a new combination: *Metacephalozia crispata*. Taking into account the limited amount of information on this taxon, we provide the corresponding figures for *M. crispata* (**Figures 6**–**8**).

*M. crispata* (N.Kitag.) Bakalin, Maltseva, Troitzk. *comb. nov.*

Basionym: *C. crispata* N.Kitag., J. Hattori Bot. Lab. 32: 301 1969 (Kitagawa 1969c). = *C. exigua* (Inoue) R.M. Schust. & Inoue Bull. Natl. Sci. Mus. Tokyo, n.s. 17: 162 1974; = *M. exigua* Inoue J. Hattori Bot. Lab. 37: 287 1973.

At last, we provide the generic list of Cephaloziellaceae s. str. as it is accepted in the present paper. The asterisk indicates that the genus was not studied by molecular genetic methods and that its status, as well as its scope, is not definitely known. The exclamation mark indicates that at least one species of the genus was involved in the present study and that its position within Cephaloziellaceae s. str. is now confirmed.

*Amphicephalozia R.M.Schust., Nova Hedwigia 22: 131, 1971 [1972]. The genus includes three species, and none of them are tested genetically. Type: *Amphicephalozia amplexicaulis* R.M. Schust. Nova Hedwigia 22(1–2): 133. 1971[1972].

*Cephalojonesia Grolle, Rev. Bryol. Lichénol. 37: 763 1971. The genus is monospecific. Type: *Cephalojonesia incuba* Grolle & Vanden Berghen Rev. Bryol. Lichénol. 37: 764, pl. 1–2 1970 [1971].

!*Cephaloziella* (Spruce) Schiffn., Hepat. (Engl.-Prantl): 98 1893. Basionym: *Cephalozia* subgen. *Cephaloziella* Spruce, Cephalozia: 62 1882. The exact number of species is unknown due to results obtained in the present paper; this genus should be split into several genera. Type: *C. divaricata* (Sm.) Schiffn., Hepat. (Engl.-Prantl): 99 1893.

!*Cephalomitrion* R.M.Schust., Nova Hedwigia 61: 550 1995. The genus is monospecific. Type: *Cephalomitrion aterrimum* (Steph.) R.M. Schust., Nova Hedwigia 61 (3/4): 554 1995.

!*Cephaloziopsis* (Spruce) Schiffn., Hepat. (Engl.-Prantl): 85 1893. Basionym: *Jungermannia* sect. *Cephaloziopsis* Spruce, Trans. & Proc. Bot. Soc. Edinburgh 15: 511, 1885. The genus is monospecific. Type: *C. intertexta* (Gottsche) R.M.Schust., Nova Hedwigia 22: 183 1971 [1972].

!*Cylindrocolea* R.M.Schust., Bull. Natl. Sci. Mus. Tokyo 12: 664 1969. The genus includes 18 recognized taxa, and only a few of them were tested genetically. Type: *C. chevalieri* (Steph.) R.M.Schust., Bull. Natl. Sci. Mus. Tokyo (n.ser.) 12 (3): 666 1969.

!*Douiniella* Bakalin, Maltseva, Troitzk. (the present paper). The genus is confirmed as monospecific (may be bi-specific, but it requires additional studies). Type: *D. spinicaulis* Bakalin, Maltseva, Troitzk. (the present paper).

*Gymnocoleopsis (R.M.Schust.) R.M.Schust., Phytologia 39: 243, 1978. Basionym: *Gymnocolea* subgen. *Gymnocoleopsis* R.M.Schust., Bryologist 70: 111, 1967. The genus includes two recognized taxa, but none of them were tested genetically. Type: *Gymnocoleopsis multiflora* (Steph.) R.M. Schust. Phytologia 39: 243. 1978. (=*Gymnocoleopsis cylindriformis* (Mitt.) R.M. Schust., J. Hattori Bot. Lab. 78: 126, 1995).

!*Kymatocalyx* Herzog, Memoranda Soc. Fauna Fl. Fenn. 25: 55, 1950. The genus includes four recognized taxa, and one of them was tested genetically. Type: *K. stolonifer* Herzog Memoranda Soc. Fauna Fl. Fenn. 25: 55 1950. (=*K. dominicensis* (Spruce) Váňa, Österr. Bot. Z. 118 (5): 575 1970).

!*Metacephalozia* Inoue J. Hattori Bot. Lab. 37: 287 1973. The genus is monospecific: *M. crispata* (N.Kitag.) Bakalin, Maltseva, Troitzk. (the present paper). The generic status is confirmed in the present paper.

!*Prionolobus* (Spruce) Schiffn. Hepaticae … aus Engler-Prantl 98 1893. The genus is confirmed as monospecific (actually it may include more taxa, but it requires additional studies). Type: *P. turneri* (Hook.) Schiffn. Hepaticae … aus Engler-Prantl 98 1893.

## 5 Conclusions

In general, the attempt to construct Cephaloziellaceae trees based on five genes revealed how little information is available on this family. A number of genera have not yet been studied genetically. For *Cephalomitrion* (characterized by the peculiar structure of seta 4 + 8), the data do not give an unambiguous answer to the question of whether it should be included in *Cylindrocolea* or considered as a separate genus. The resulting tree topologies highlight the need for critical studies of other genera within Cephaloziellaceae s. str. In contrast, it may be assumed that the reduction of *Dichiton* to the synonyms of *Cephaloziella* (such as *Cephaloziella* subg. *Dichiton* (Mont.) Müll.Frib.) may be quite premature, given the large genetic distances between subgenera that have already been studied.

As shown by the present study, the Cephaloziellaceae system recognized in the World Liverwort Checklist (Söderström et al., 2016) does not perfectly reflect phylogenetic relationships and should be corrected. Some of the widely recognized genera may not deserve independence (*Kymatocalyx*, *Cephalomitrion*). At the same time, some species groups of the large genus *Cephaloziella* should be segregated into distinct genera. Some of them are simply genera described a long time ago and undeservedly forgotten. Given that molecular data are available for only less than 15% of the recognized species within Cephaloziellaceae, a large number of new “surprises” can be expected in further studies of this family of the smallest higher plants.

## Data availability statement

The datasets presented in this study can be found in online repositories. The names of the repository/repositories and accession number(s) can be found in the article/**Supplementary Material**.

## Author contributions

VB: Funding acquisition, Investigation, Writing – review & editing, Data curation, Project administration, Supervision, Validation, Writing – original draft. YM: Formal Analysis, Investigation, Methodology, Visualization, Writing – original draft. KK: Investigation, Resources, Visualization, Writing – review & editing. VN: Formal Analysis, Investigation, Resources, Writing – review & editing. SC: Funding acquisition, Investigation, Resources, Writing – review & editing. AT: Data curation, Formal Analysis, Methodology, Supervision, Validation, Writing – original draft, Writing – review & editing.
