# Supplementary material for: New insight into the taxonomy of Cephaloziellaceae (Marchantiophyta): the family of the smallest higher plants on Earth
Source: Front Plant Sci. 2024 Feb 29;15:1326810. doi: 10.3389/fpls.2024.1326810 (PMC10937528; doi:10.3389/fpls.2024.1326810)
Supplement: Supplementary file 1 [file Table_1.docx]

Supplementary Material

New insight into the taxonomy of Cephaloziellaceae (Marchantiophyta) – the family of the smallest higher plants on Earth

Vadim A. Bakalin*, Yulia D. Maltseva, Ksenia G. Klimova, Van Sinh Nguyen, Seung Se Choi*, Aleksey V. Troitsky

*** Correspondence:** Vadim A. Bakalin: vabakalin@gmail.com; Seung Se Choi: hepaticae@jbnu.ac.kr

**Supplementary Table 1.** The list of voucher details and GenBank accession numbers for the specimens used in phylogenetic reconstructions in present paper. DNA regions sequenced in this work are highlighted in bold.

| Initial species name | Accepted name | Label | GenBank accession number | | | | |
| --- | --- | --- | --- | --- | --- | --- | --- |
|  |  |  | ITS1–2 | *trn*L–F | *trn*G-intron | *rbc*L | *psb*A |
| *Anastrophyllopsis subcomplicata* (Lehm. et Lindenb.) Váňa et L.Söderstr. | *Anastrophyllopsis subcomplicata* (Lehm. et Lindenb.) Váňa et L.Söderstr. | Australia, D.A. Meagher & N. Klazenga 07-142 (F) |  |  |  |  | KF942484 |
| *Anastrophyllopsis subcomplicata* (Lehm. et Lindenb.) Váňa et L.Söderstr. | *Anastrophyllopsis subcomplicata* (Lehm. et Lindenb.) Váňa et L.Söderstr. | Australia: Tasmania, B. Shaw 6385 (DUKE) |  |  |  |  | KF851961 |
| *Anastrophyllum assimile* (Mitt.) Steph. | *Anastrophyllum assimile* (Mitt.) Steph. | Korea, Gang won Province, V.A. Bakalin, Kor-5-12-11 (VBGI) | KF836652 | KF836662 | KF836641 |  |  |
| *Anastrophyllum ellipticum* Inoue | *Anastrophyllum ellipticum* Inoue | Russia, Altai Territory, Yu. Mamontov, 330/2 (KPABG) | KF836654, KF836632 | KF836664 | KF836642 |  |  |
| *Anastrophyllum michauxii* (F.Weber) H.Buch | *Anastrophyllum michauxii* (F.Weber) H.Buch | USA, North Carolina, Clingman's Dome, M. Sargent, s.n. (ABSH) |  | AY507519 |  | AY507390 |  |
| *Anastrophyllum minutum* (Schreb. ex Cranz) R.M. Schust. | *Sphenolobus minutus* (Schreb. ex Cranz) Berggr. | Norway, Spitsbergen, Hentschel, Bryo 0421 (GOET) |  |  |  | DQ312475 |  |
| *Cephalomitrion aterrimum* (Steph.) R.M.Schust. | *Cephalomitrion aterrimum* (Steph.) R.M.Schust. | New Zealand, Engel & von Konrat, 28545 (F) |  |  |  | KF852368 | KF851926 |
| *Cephaloziella* aff. *kiaeri* (Austin) Douin | *Cylindrocolea kiaeri* (Austin) Váňa | South Korea, Jeollabuk-do, V.A. Bakalin & S.S. Choi, Kor-72-3-19 (VBGI) |  | **OP205287** | **OP205272** |  |  |
| *Cephaloziella arctogena* (R.M.Schust.) Konstant. | *Cephaloziella arctogena* (R.M.Schust.) Konstant. | Russia, Buryatiya Rep., O. Afonina, 45907 (KPABG) |  | JX630045 |  |  |  |
| *Cephaloziella aspericaulis* Jørg. | *Cephaloziella konstantinovae* Mamontov & Vilnet | Russia, Buryatiya Rep., O. Afonina, 5307 (KPABG) |  | JX630044 |  |  |  |
| *Cephaloziella aspericaulis* Jørg. | *Cephaloziella konstantinovae* Mamontov & Vilnet | Russia, Republic of Buryatia, O. Afonina, A4006 (KPABG) | KF471666 | KF471664 |  |  |  |
| *Cephaloziella crispata* N.Kitag. | *Cephaloziella crispata* N.Kitag. | Northwest Vietnam, Sơn La Province, V.A. Bakalin & K.G. Klimova, V-18-4-19 (VBGI) |  | **OP205288** | **OP205273** | **OP205308** | **OP180074** |
| *Cephaloziella divaricata* (Sm.) Schiffn. | *Cephaloziella divaricata* (Sm.) Schiffn. | Russia, Republic of Buryatia, O. Afonina, A07408 (KPABG) | JX629921 | JX630050 |  |  |  |
| *Cephaloziella divaricata* (Sm.) Schiffn. | *Cephaloziella divaricata* (Sm.) Schiffn. | Lithuania, The Curonian Spit, N. Kalinauskait, #87 (KPABG) |  | KF805922 |  |  |  |
| *Cephaloziella divaricata* (Sm.) Schiffn. | *Cephaloziella divaricata* (Sm.) Schiffn. | Czech Republic, P. Sova, Aug-2008 (DUKE) |  | KJ802094 | KJ802056 | KF852399 | KF851965 |
| *Cephaloziella divaricata* (Sm.) Schiffn. | *Cephaloziella divaricata* (Sm.) Schiffn. | Russia, Russian Far East, Sakhalin Province, Sakhalin Island, V. Bakalin, S-24-21-09 (KPABG) | KF805881 | KF805923 |  |  |  |
| *Cephaloziella divaricata* (Sm.) Schiffn. | *Cephaloziella divaricata* (Sm.) Schiffn. | Russia, Russian Far East, Kamchatka Territory, V.A. Bakalin, K-57-6-15 (VBGI) |  | MT476333 | MT482532 |  |  |
| *Cephaloziella divaricata* (Sm.) Schiffn. | *Cephaloziella divaricata* (Sm.) Schiffn. | Germany, Hentschel, Bryo 01159 (GOET) |  |  |  | DQ312481 | AM396180 |
| *Cephaloziella divaricata* (Sm.) Schiffn. | *Cephaloziella divaricata* (Sm.) Schiffn. | Russia, Russian Far East, Khabarovsk Territory, V.A. Bakalin & K.G. Klimova, Kh-51-2-19 (VBGI) | **OP082396** | **OP205289** | **OP205274** | **OP205309** |  |
| *Cephaloziella elachista* (J.B.Jack ex Gottsche et Rabenh.) Schiffn. | *Cephaloziella elachista* (J.B.Jack ex Gottsche et Rabenh.) Schiffn. | Russia, Khanty-Mansi Aotonomous Area, OP1BH607/7 (KPABG) | JX629918 | JX630047 |  |  |  |
| *Cephaloziella elachista* (J.B.Jack ex Gottsche et Rabenh.) Schiffn. | *Cephaloziella elachista* (J.B.Jack ex Gottsche et Rabenh.) Schiffn. | Russia, Tuva Rep., T. Otnyukova, 101195 (VBGI) |  | KF805929 |  |  |  |
| *Cephaloziella elachista* (J.B.Jack ex Gottsche et Rabenh.) Schiffn. | *Cephaloziella elachista* (J.B.Jack ex Gottsche et Rabenh.) Schiffn. | Russia, Khanty-Mansi Autonomous Area, G. Kukurichkin, T2VNPP104a |  | KF805930 |  |  |  |
| *Cephaloziella granatensis* (J.B.Jack ex Steph.) Fulford | *Cephaloziella granatensis* (J.B.Jack ex Steph.) Fulford | Panama, Dauphin et al., 1548 (GOET) |  |  |  | KC184715 | KC184783 |
| *Cephaloziella grimsulana* (J.B.Jack ex Gottsche et Rabenh.) Lacout. | *Cephaloziella grimsulana* (J.B.Jack ex Gottsche et Rabenh.) Lacout. | Russia, Chukotka Territory, O. Afonina, 77 (LE) |  | KF805910 |  |  |  |
| *Cephaloziella grimsulana* (J.B.Jack ex Gottsche et Rabenh.) Lacout. | *Cephaloziella grimsulana* (J.B.Jack ex Gottsche et Rabenh.) Lacout. | Russia, Krasnoyarsk Territory, Novaya Zemlya, I. Bolshevik, I. Safronova, 77 (LE) |  | KF805909 |  |  |  |
| *Cephaloziella hirta* (Steph.) R.M.Schust. | *Cephaloziella hirta* (Steph.) R.M.Schust. | Australia, Streimann 59793 (NY) |  | KF943000, KJ802079 | KF942864 | DQ439682 | AY607933 |
| *Cephaloziella kiaeri* (Austin) Douin | *Cylindrocolea kiaeri* (Austin) Váňa | Korea, South Korea, V.A. Bakalin, Kor-16-13-08 (KPABG) |  | KF805931 |  |  |  |
| *Cephaloziella kiaeri* (Austin) Douin | *Cylindrocolea kiaeri* (Austin) Váňa | Korea, South Korea, V.A. Bakalin, Kor-13-13-08 (KPABG) |  | KF805932 |  |  |  |
| *Cephaloziella kiaeri* (Austin) Douin | *Cylindrocolea kiaeri* (Austin) Váňa | Korea, South Korea, V.A. Bakalin, Kor-2-14-09 (KPABG) | KF805887 | KF805933 |  |  |  |
| *Cephaloziella kiaeri* (Austin) Douin | *Cylindrocolea kiaeri* (Austin) Váňa | Japan, Bryophyte Selecta Exsiccatae №1218 (KPABG) | KF805886 | KF805934 |  |  |  |
| *Cephaloziella kiaeri* (Austin) Douin | *Cylindrocolea kiaeri* (Austin) Váňa | Borneo, Kinabalu, N. Konstantinova, K-8-4 (KPABG) | KF805888 | KF805935 |  |  |  |
| *Cephaloziella kiaeri* (Austin) Douin | *Cylindrocolea kiaeri* (Austin) Váňa | Russia, Russian Far East, Primorsky Territory, Khasansky District, V.A. Bakalin, P-3-25-07 (VBGI) |  | KF805936 |  |  |  |
| *Cephaloziella kiaeri* (Austin) Douin | *Cylindrocolea kiaeri* (Austin) Váňa | South Korea, Chungcheongnam-do, V.A. Bakalin & S.S. Choi, Kor-84-2-19 (VBGI) | **OP082397** | **OP205290** | **OP205275** |  | **OP205301** |
| *Cephaloziella massalongi* (Spruce) Müll.Frib. | *Cephaloziella massalongi* (Spruce) Müll.Frib. | Korea, South Korea, Hepaticae Korea Exsiccatae Fasc. I №51 (KPABG) |  | KF805917 |  |  |  |
| *Cephaloziella massalongi* (Spruce) Müll.Frib. | *Cephaloziella massalongi* (Spruce) Müll.Frib. | Korea, South Korea, V.A. Bakalin, Kor-10-7-09 (KPABG) |  | KF805918 |  |  |  |
| *Cephaloziella microphylla* (Steph.) Douin | *Cephaloziella microphylla* (Steph.) Douin | Korea, South Korea, Hepaticae Korea Exsiccatae Fasc.II №23 (KPABG) | KF805889 | KF805937 |  |  |  |
| *Cephaloziella microphylla* (Steph.) Douin | *Cephaloziella microphylla* (Steph.) Douin | Korea, South Korea, Hepaticae Korea Exsiccatae Fasc.I №29 (KPABG) | KF805890 | KF805938 |  |  |  |
| *Cephaloziella microphylla* (Steph.) Douin | *Cephaloziella microphylla* (Steph.) Douin | Thailand, Schaefer-Verwimp and Verwimp 16291 (GOET) |  |  |  | KC184716 | KC184784 |
| *Cephaloziella polystratosa* (R.M.Schust. et Damsh.) Konstant. | *Cephaloziella polystratosa* (R.M.Schust. et Damsh.) Konstant. | Russia, Buryatiya Rep., O. Afonina, A1810 (KPABG) | KF471665 | JX630046 |  |  |  |
| *Cephaloziella rubella* (Nees) Warnst. | *Cephaloziella rubella* (Nees) Warnst. | Russia, Altai Territory, Yu. Mamontov, 214-14 (KPABG) | KF805885 | KF805927 |  |  |  |
| *Cephaloziella rubella* (Nees) Warnst. | *Cephaloziella rubella* (Nees) Warnst. | Russia, Murmansk Prov., E. Golovina, 3/4 (KPABG) |  | KF805928 |  |  |  |
| *Cephaloziella rubella* (Nees) Warnst. | *Cephaloziella rubella* (Nees) Warnst. | Russia, Altai Territory, Yu. Mamontov, 330-5-1 (KPABG) | KF853559 | KF853560 |  |  |  |
| *Cephaloziella rubella* (Nees) Warnst. | *Cephaloziella elachista* (J.B.Jack ex Gottsche et Rabenh.) Schiffn. | Russia, Khanty-Mansi Aotonomous Area, G. Kukurichkin, OP1BH607/1 (KPABG) | JX629919 | JX630048 |  |  |  |
| *Cephaloziella* sp. | *Cephaloziella* sp. | Russia, Buryatiya Rep., O. Afonina, A4805 (KPABG) | JX629920 | JX630049 |  |  |  |
| *Cephaloziella* sp. | *Cephaloziella konstantinovae* Mamontov & Vilnet | Russia, Trans-Baikal Territory, Yu. Mamontov, 5307a (KPABG) | KF805884 | KF805926 |  |  |  |
| *Cephaloziella* sp. | *Cephaloziella* sp. | Russia, Russian Far East, Khabarovsk Territory, V.A. Bakalin, Kh-72-25-09 (KPABG) | KF805895 |  |  |  |  |
| *Cephaloziella* sp. | *Cephaloziella* sp. | China, Yunnan Province, V.A. Bakalin & W.Z. Ma, C-82-11-18 (VBGI) | **OP082398** | **OP205291** | **OP205276** |  | **OP205302** |
| *Cephaloziella spinicaulis* Douin | *Cephaloziella spinicaulis* Douin | Japan, Deguchi, Ex 5 (1998) IV 119 (KPABG) | JX629922 | JX630043 |  |  |  |
| *Cephaloziella spinicaulis* Douin | *Cephaloziella spinicaulis* Douin | Russia, Russian Far East, Primorsky Territory, V.A. Bakalin, P-63-3-08 (KPABG) | KF805896 | KF805947 |  |  |  |
| *Cephaloziella spinicaulis* Douin | *Cephaloziella spinicaulis* Douin | Japan, Honshu, Hiroshima, Shiwa, H. Masuzaki, 385 (HIRO) |  |  |  | AB476561 |  |
| *Cephaloziella spinicaulis* Douin | *Cephaloziella spinicaulis* Douin | Japan, Deguchi 119 (GOET) |  |  |  | KC184717 |  |
| *Cephaloziella spinicaulis* Douin | *Cephaloziella spinicaulis* Douin | South Korea, Jeollabuk-do, V.A. Bakalin & S.S. Choi, Kor-71-2-19 (VBGI) |  | **OP205292** | **OP205277** |  | **OP205303** |
| *Cephaloziella stellulifera* (Taylor ex Carrington et Pearson) Croz. | *Cephaloziella stellulifera* (Taylor ex Carrington et Pearson) Croz. | Netherlands, N. Konstantinova, 1e-2-99 (KPABG) | JX629923 | JX630042 |  |  |  |
| *Cephaloziella stellulifera* (Taylor ex Carrington et Pearson) Croz. | *Cephaloziella stellulifera* (Taylor ex Carrington et Pearson) Croz. | USA, California, Doyle 11250 (GOET) |  |  |  | KC184718 | KC184786 |
| *Cephaloziella subdentata* Warnst. | *Cephaloziella spinigera* (Lindb.) Warnst. | Russia, Russian Far East, Kamchatka Territory, V.A. Bakalin, 98-4-01-VB, 104011 (KPABG) | KF805865 | KF805897 |  |  |  |
| *Cephaloziella subdentata* Warnst. | *Cephaloziella spinigera* (Lindb.) Warnst. | Russia, Murmansk Province, N. Konstantinova, 182-3 (KPABG) | KF805864 | KF805898 |  |  |  |
| *Cephaloziella subdentata* Warnst. | *Cephaloziella spinigera* (Lindb.) Warnst. | Russia, Russian Far East, Kamchatka Territory, V.A. Bakalin, 77-14-01-VB, 103933 (KPABG) | KF805866 | KF805899 |  |  |  |
| *Cephaloziella tahora* Bever. & Glenny | *Cephaloziella tahora* Bever. & Glenny | New Zealand, P Beveridge MV10a H013199 (WELT) |  |  |  | KT705315 |  |
| *Cephaloziella turneri* (Hook.) Müll.Frib. | *Cephaloziella turneri* (Hook.) Müll.Frib. | Russia, Krasnodar Territory, M. Ignatov, E. Ignatova, 02-24a (KPABG) | KF805892 | KF805944 |  |  |  |
| *Cephaloziella turneri* (Hook.) Müll.Frib. | *Cephaloziella turneri* (Hook.) Müll.Frib. | Russia, Krasnodar Territory, N. Konstantonova, K370-4-08, 112561 (KPABG) | KF805893 | KF805945 |  |  |  |
| *Cephaloziella turneri* (Hook.) Müll.Frib. | *Cephaloziella turneri* (Hook.) Müll.Frib. | Russia, Krasnodar Territory, N. Konstantonova, K346-8-11, 114984 (KPABG) | KF805894 |  |  |  |  |
| *Cephaloziella turneri* (Hook.) Müll.Frib. | *Cephaloziella turneri* (Hook.) Müll.Frib. | USA, California, J.R. Shevock, 27856 (GOET) |  |  |  | KC184719 | KC184787 |
| *Cephaloziella varians* (Gottsche) Steph. | *Cephaloziella varians* (Gottsche) Steph. | Russia, Altai Territory, Yu. Mamontov, 219/5 (KPABG) | KF805876, KF805862 | KF805912 |  |  |  |
| *Cephaloziella varians* (Gottsche) Steph. | *Cephaloziella varians* (Gottsche) Steph. | Russia, Russian Far East, Kamchatka Province, V.A. Bakalin, K-105-5-03 (KPABG) |  | JX630051 |  |  |  |
| *Cephaloziella varians* (Gottsche) Steph. | *Cephaloziella varians* (Gottsche) Steph. | Russia, Trans-Baikal Territory, O. Afonina, A2410 (KPABG) | KF805867 | KF805900 |  |  |  |
| *Cephaloziella varians* (Gottsche) Steph. | *Cephaloziella varians* (Gottsche) Steph. | Russia, Trans-Baikal Territory, O. Afonina, A6010b (KPABG) | KF805868 | KF805901 |  |  |  |
| *Cephaloziella varians* (Gottsche) Steph. | *Cephaloziella varians* (Gottsche) Steph. | Russia, Trans-Baikal Territory, O. Afonina, A5610/4 (KPABG) | KF805869 | KF805902 |  |  |  |
| *Cephaloziella varians* (Gottsche) Steph. | *Cephaloziella varians* (Gottsche) Steph. | Russia, Trans-Baikal Territory, O. Afonina, A3010 (KPABG) | KF805870 | KF805903 |  |  |  |
| *Cephaloziella varians* (Gottsche) Steph. | *Cephaloziella varians* (Gottsche) Steph. | Russia, Murmansk Province, N.A. Konstantinova, K 8-2-12 (KPABG) | KF805871, KF805861 | KF805904 |  |  |  |
| *Cephaloziella varians* (Gottsche) Steph. | *Cephaloziella varians* (Gottsche) Steph. | Russia, Magadan Province, V.A. Bakalin, Mag-7-40-10 (KPABG) | KF805872 | KF805905 |  |  |  |
| *Cephaloziella varians* (Gottsche) Steph. | *Cephaloziella varians* (Gottsche) Steph. | Russia, Khanty-Mansi Autonomous Area, G. Kukurichkin, T2VNPP104b (KPABG) |  | KF805906 |  |  |  |
| *Cephaloziella varians* (Gottsche) Steph. | *Cephaloziella varians* (Gottsche) Steph. | Russia, Republic of Karachaevo-Cherkessia, N.A. Konstantinova, K517-2-05 (KPABG) |  | KF805907 |  |  |  |
| *Cephaloziella varians* (Gottsche) Steph. | *Cephaloziella varians* (Gottsche) Steph. | Russia, Republic of Karachaevo-Cherkessia, N.A. Konstantinova, K515-1-05 (KPABG) |  | KF805908 |  |  |  |
| *Cephaloziella varians* (Gottsche) Steph. | *Cephaloziella varians* (Gottsche) Steph. | Russia, Russian Far East, Sakhalin Province, Sakhalin Island, V.A. Bakalin, S-33-2a-09 (KPABG) | KF805875 | KF805911 |  |  |  |
| *Cephaloziella varians* (Gottsche) Steph. | *Cephaloziella varians* (Gottsche) Steph. | Russia, Russian Far East, Primorsky Territory, Yu. Mamontov, 185-5-10 (KPABG) |  | KF805913 |  |  |  |
| *Cephaloziella varians* (Gottsche) Steph. | *Cephaloziella varians* (Gottsche) Steph. | Norway, Svalbard, N.A. Konstantinova, K78-1-06 (KPABG) | KF805877, KF805863 | KF805914 |  |  |  |
| *Cephaloziella varians* (Gottsche) Steph. | *Cephaloziella varians* (Gottsche) Steph. | Norway, Svalbard, N.A. Konstantinova, K115-02 (KPABG) | KF805878 | KF805915 |  |  |  |
| *Cephaloziella varians* (Gottsche) Steph. | *Cephaloziella varians* (Gottsche) Steph. | Russia, Russian Far East, Sakhalin Province, Sakhalin Island, V.A. Bakalin, S-58-3-09 (KPABG) |  | KF805916 |  |  |  |
| *Cephaloziella varians* (Gottsche) Steph. | *Cephaloziella varians* (Gottsche) Steph. | Russia, Trans-Baikal Territory, O. Afonina, A05307 (KPABG) |  | KF805919 |  |  |  |
| *Cephaloziella varians* (Gottsche) Steph. | *Cephaloziella varians* (Gottsche) Steph. | Norway, Svalbard, N.A. Konstantinova, K130-2-04 (KPABG) | KF805880 | KF805920 |  |  |  |
| *Cephaloziella varians* (Gottsche) Steph. | *Cephaloziella varians* (Gottsche) Steph. | Russia, Russian Far East, Khabrovsk Territory, V.A. Bakalin, Kh-34-10-08 (KPABG) |  | KF805921 |  |  |  |
| *Cephaloziella varians* (Gottsche) Steph. | *Cephaloziella varians* (Gottsche) Steph. | New Zealand, Engel, 21964 (F) |  |  |  | DQ439689 | AY607953 |
| *Cephaloziella varians* (Gottsche) Steph. | *Cephaloziella varians* (Gottsche) Steph. | Russia, Russian Far East, Kamchatka Territory, V.A. Bakalin & K.G. Klimova, K-102-18-21 |  | **OP205293** | **OP205278** |  |  |
| *Cylindrocolea kiaeri* (Austin) Váňa | *Cylindrocolea kiaeri* (Austin) Váňa | Northeast Vietnam, Hà Giang Province, V.A. Bakalin & K.G. Klimova, V-15-4-20 (VBGI) |  | **OP205295** | **OP205280** | **OP205311** | **OP205305** |
| *Cylindrocolea kiaeri* (Austin) Váňa | *Cylindrocolea kiaeri* (Austin) Váňa | North Vietnam, Lao Cai Province, V.A. Bakalin & K.G. Klimova, V-17-35-18 (VBGI) |  | **OP205294** | **OP205279** | **OP205310** | **OP205304** |
| *Cephaloziopsis exigua* (Inoue) R.M. Schust. & Inoue | *Metacephalozia crispata* Bakalin, Maltseva, Troitzk. | Northeast Vietnam, Cao Bằng Province, V.A. Bakalin & K.G. Klimova, V-23-11-20 (VBGI) | **OP082399** | **OP205286** | **OP205271** | **OP205307** | **OP180073** |
| *Cephaloziopsis intertexta* (Gottsche) R.M.Schust. | *Cephaloziopsis intertexta* (Gottsche) R.M.Schust. | Bolivia, Linneo et al., 424 (GOET) |  |  |  | KC184720 | KC184788 |
| *Chaetophyllopsis whiteleggei* (Carrington et Pearson) R.M.Schust. ex Hamlin | *Chaetophyllopsis whiteleggei* (Carrington et Pearson) R.M.Schust. ex Hamlin | Australia, Western Australia, J.A. Curnow, 4804 (F) |  | AY463553 |  | AY462292, KF852333 | KF851893 |
| *Cylindrocolea obtusifolia* Fulford | *Cylindrocolea obtusifolia* Fulford | Brazil, 12991 (LE) |  | KF805940 |  |  |  |
| *Cylindrocolea recurvifolia* (Steph.) Inoue | *Cylindrocolea recurvifolia* (Steph.) Inoue | Japan, Kyushu Island, Exsiccatae Bryophytes of Asia Fasc.14 №344 (KPABG) |  | JX630061 |  |  |  |
| *Cylindrocolea recurvifolia* (Steph.) Inoue | *Cylindrocolea recurvifolia* (Steph.) Inoue | Japan, Kyushu Island, Exsiccatae Bryophytes of Asia Fasc.14 №344 (KPABG) | KF805891 | KF805939 |  |  |  |
| *Cylindrocolea recurvifolia* (Steph.) Inoue | *Cylindrocolea recurvifolia* (Steph.) Inoue | Korea, South Korea, Hepaticae Korea Exsiccatae Fasc. II №92 (KPABG) |  | KF805941 |  |  |  |
| *Cylindrocolea recurvifolia* (Steph.) Inoue | *Cylindrocolea recurvifolia* (Steph.) Inoue | Korea, South Korea, V.A. Bakalin, Kor-16-27-11 (KPABG) |  | KF805942 |  |  |  |
| *Cylindrocolea recurvifolia* (Steph.) Inoue | *Cylindrocolea recurvifolia* (Steph.) Inoue | Japan, Deguchi, 36641 (HIRO) |  |  |  | AM392306 |  |
| *Cylindrocolea recurvifolia* (Steph.) Inoue | *Cylindrocolea recurvifolia* (Steph.) Inoue | Japan, T. Yamaguchi (F) |  |  |  | KF852297 | KF851848 |
| *Cylindrocolea recurvifolia* (Steph.) Inoue | *Cylindrocolea recurvifolia* (Steph.) Inoue | Japan, Honshu Island, Hiroshima, Miyajima, H. Masuzaki, 679 (HIRO) |  |  |  | AB476564 |  |
| *Cylindrocolea recurvifolia* (Steph.) Inoue | *Cylindrocolea recurvifolia* (Steph.) Inoue | Japan, Yamaguchi 28949 (GOET) |  |  |  | KC184722 | KC184790 |
| *Cylindrocolea recurvifolia* (Steph.) Inoue | *Cylindrocolea recurvifolia* (Steph.) Inoue | North Vietnam, Lai Châu Province, V.A. Bakalin & K.G. Klimova, V-9-2-18 (VBGI) |  | **OP205296** | **OP205281** |  |  |
| *Cylindrocolea recurvifolia* (Steph.) Inoue | *Cylindrocolea recurvifolia* (Steph.) Inoue | South Korea, Gyeongsangnam-do, V.A. Bakalin & S.S. Choi, Kor-70-13-19 (VBGI) |  | **OP205297** | **OP205282** | **OP205312** |  |
| *Gottschelia schizopleura* (Spruce) Grolle | *Gottschelia schizopleura* (Spruce) Grolle | Malaysia, Pahang, D.J. Long 36922 (E ) |  | KJ802085 |  | KF852362 | KF851920 |
| *Gottschelia schizopleura* (Spruce) Grolle | *Gottschelia schizopleura* (Spruce) Grolle | Reunion, Schaefer-Verwimp, 19823 |  |  |  | FJ984938 |  |
| *Gottschelia schizopleura* (Spruce) Grolle | *Gottschelia schizopleura* (Spruce) Grolle | Reunion, Gradstein, 12032 |  |  |  | FJ984939 |  |
| *Gottschelia schizopleura* (Spruce) Grolle | *Gottschelia schizopleura* (Spruce) Grolle | Madagascar, Ah-Peng R96 (GOET) |  |  |  | FJ984940 | KC184793 |
| *Gottschelia schizopleura* (Spruce) Grolle | *Gottschelia schizopleura* (Spruce) Grolle | Indonesia, Gradstein, 12065 (GOET) |  |  |  | FJ984941 |  |
| *Gottschelia schizopleura* (Spruce) Grolle | *Gottschelia schizopleura* (Spruce) Grolle | Indonesia, Gradstein, 12064 (GOET) |  |  |  | FJ984942 |  |
| *Gottschelia schizopleura* (Spruce) Grolle | *Gottschelia schizopleura* (Spruce) Grolle | Sri Lanka, Schaefer-Verwimp, 5488II |  |  |  | FJ984943 |  |
| *Gottschelia schizopleura* (Spruce) Grolle | *Gottschelia schizopleura* (Spruce) Grolle | Malaysia, Schaefer-Verwimp 18695 (GOET) |  |  |  | FJ984944 |  |
| *Herzogobryum atrocapillum* (Hook.f. et Taylor) Grolle | *Herzogobryum atrocapillum* (Hook.f. et Taylor) Grolle | South Africa, Marion Island, R. Ochyra 1402/01 (DUKE) |  |  | KF942933 |  |  |
| *Herzogobryum teres* (Carrington & Pearson) Grolle | *Herzogobryum teres* (Carrington & Pearson) Grolle | New Zealand, Braggins 92/102 (F) |  |  |  |  | AY607951 |
| *Herzogobryum vermiculare* (Schiffn.) Grolle | *Herzogobryum vermiculare* (Schiffn.) Grolle | French Southern and Antarctic Lands, Iles Kerguelen, R. Ochyra 1011/06 (DUKE) |  | KF943047 | KF942886 | KF943587 | KF942562 |
| *Hygrobiella laxifolia* (Hook.) Spruce | *Hygrobiella laxifolia* (Hook.) Spruce | Russia, V.A. Bakalin 61 (GOET) |  |  |  |  | KC184794 |
| *Hygrobiella laxifolia* (Hook.) Spruce | *Hygrobiella laxifolia* (Hook.) Spruce | Russia, V.A. Bakalin (F) |  |  |  | KF852320 |  |
| *Hygrobiella laxifolia* (Hook.) Spruce | *Hygrobiella laxifolia* (Hook.) Spruce | Russia, N.A. Konstantinova 31-2-03 (F) |  |  |  |  | KF942489 |
| *Hygrobiella laxifolia* (Hook.) Spruce | *Hygrobiella laxifolia* (Hook.) Spruce | Russia, Russian Far East, Kamchatka Territory, V.A. Bakalin & K.G. Klimova, K-85-3b-21 |  | **OP205299** | **OP205284** |  |  |
| *Hygrobiella nishimurae* N.Kitag. | *Hygrobiella nishimurae* N.Kitag. | Japan, Shikoku Island, Kochi Prefecture, V.A. Bakalin, J-11-42-15 (KPABG, VGBI) | MH580594 | MH580592 | MH580603 |  |  |
| *Hygrobiella squamosa* Bakalin et Vilnet | *Hygrobiella squamosa* Bakalin et Vilnet | Russia, Sakhalin Province, Kuril Islands, Iturup Island, V.A. Bakalin, K-12-8a-07 (VLA), duplicate 115587 (KPABG) |  | KF008585 | KF008649 |  |  |
| *Hygrobiella squamosa* Bakalin et Vilnet | *Hygrobiella squamosa* Bakalin et Vilnet | Russia, Sakhalin Province, Kuril Islands, Kunashir Island, V.A. Bakalin, K-37-17-06 (VLA), duplicate 115591 (KPABG) |  | KF008586 |  |  |  |
| *Hygrobiella squamosa* Bakalin et Vilnet | *Hygrobiella squamosa* Bakalin et Vilnet | Russia, Commander Islands, Medny Island, V.A. Bakalin, K-48-6-04 (VLA), duplicate 106663 (KPABG) |  |  | KF008650 |  |  |
| *Hygrobiella squamosa* Bakalin et Vilnet | *Hygrobiella squamosa* Bakalin et Vilnet | Russia, Russian Far East, Kamchatka Territory, V.A. Bakalin, K-19-9-21 |  | **OP205300** | **OP205285** |  | **OP205306** |
| *Kymatocalyx dominicensis* (Spruce) Váňa | *Kymatocalyx dominicensis* (Spruce) Váňa | Brazil, Schaefer-Verwimp, 12812 (LE) |  | KF805943 |  |  |  |
| *Kymatocalyx dominicensis* (Spruce) Váňa | *Kymatocalyx dominicensis* (Spruce) Váňa | Guadeloupe, Schaefer-Verwimp and Verwimp 22451 (GOET) |  |  |  | KC184726 | KC184795 |
| *Nothogymnomitrion erosum* (Carrington et Pearson) R.M.Schust. | *Nothogymnomitrion erosum* (Carrington et Pearson) R.M.Schust. | Australia, Streimann, 53475 (JE) | GQ900006 | GQ900216 |  | GQ900318 | GQ900110 |
| *Nothogymnomitrion erosum* (Carrington et Pearson) R.M.Schust. | *Nothogymnomitrion erosum* (Carrington et Pearson) R.M.Schust. | New Zealand, Engel & von Konrat s.n. (F) |  | KJ802089 |  | KF852369 | KF851928 |
| *Nothogymnomitrion erosum* (Carrington et Pearson) R.M.Schust. | *Nothogymnomitrion erosum* (Carrington et Pearson) R.M.Schust. | New Zealand, Engel, von Konrat & Braggins 24569 (F) |  |  |  | KF943548 | KF942474 |
| *Obtusifolium obtusum* (Lindb.) S.W.Arnell | *Obtusifolium obtusum* (Lindb.) S.W.Arnell | Sw-48-30-13 (VBGI) | MT504415 | MT476337 | MT482531 |  |  |
| *Obtusifolium obtusum* (Lindb.) S.W.Arnell | *Obtusifolium obtusum* (Lindb.) S.W.Arnell | Russia, Permskaya Province, N.A. Konstantinova, K-315-1-04 (KPABG) | DQ875118 |  |  |  |  |
| *Obtusifolium obtusum* (Lindb.) S.W.Arnell | *Obtusifolium obtusum* (Lindb.) S.W.Arnell | Russia, N. Konstantinova, 309-2-2000 (F) |  |  |  | KF852303 | KF851856 |
| *Obtusifolium obtusum* (Lindb.) S.W.Arnell | *Obtusifolium obtusum* (Lindb.) S.W.Arnell | Russia, Russian Far East, Kamchatka Territory, V.A. Bakalin & K.G. Klimova, K-102-2a-21 | **OP082400** | **OP205298** | **OP205283** | **OP205313** |  |
| *Oleolophozia perssonii* (H.Buch et S.W.Arnell) L.Söderstr. | *Oleolophozia perssonii* (H.Buch et S.W.Arnell) L.Söderstr. | Russia, Magadan Province, V.A. Bakalin, Mag-31-13-11, 14736 (VBGI) | MT504417 | MT476335 | MT482533 |  |  |
| *Oleolophozia perssonii* (H.Buch et S.W.Arnell) L.Söderstr. | *Oleolophozia perssonii* (H.Buch et S.W.Arnell) L.Söderstr. | Russia, Magadan Province, V.A. Bakalin, Mag-31-16-11, (VBGI) | MT504418 | MT476336 | MT482534 |  |  |
| *Protolophozia elongata* (Steph.) Schljakov | *Scapania nemorea* (L.) Grolle | Russia, Murmanskaya Province, V.A. Bakalin, 3-1-02 (KPABG) | DQ875116 | DQ875078 |  |  |  |
| *Scapania nemorea* (L.) Grolle | *Scapania nemorea* (L.) Grolle | United Kingdom, Scotland, Long, D.G. 35418 (E ) |  | KF942952 | KF942824 |  | KF942463 |
| *Scapania nemorea* (L.) Grolle | *Scapania nemorea* (L.) Grolle | USA, Illinois, Pope Co., Stotler & Crandall-Stotler s.n. [ref. no. 265] |  |  |  | AY507423 | AY507509 |
| *Scapania nemorosa* (L.) Dumort. | *Scapania nemorea* (L.) Grolle | USA, Davis, 124 (DUKE) |  | AY608143 | AY608190 | AY608039 |  |
| *Scapania nimbosa* Taylor | *Scapania nimbosa* Taylor | United Kingdom, Scotland, D.G Long & M. Flagmeier, 37028 (DUKE) |  |  |  | KF852408 | KF851976 |
| *Scapania undulata* (L.) Dumort. | *Scapania undulata* (L.) Dumort. | Finland, Nuuksio National Park, 2000 He-Nygren & Piippo, 1468 |  | AY149859 |  | AY149840 |  |
| *Scapania undulata* (L.) Dumort. | *Scapania undulata* (L.) Dumort. | USA, Shevock et al., 29009 (GOET) | JN631489 | JN631623 |  |  |  |
| *Scapania undulata* (L.) Dumort. | *Scapania undulata* (L.) Dumort. | Portugal, Schaefer-Verwimp & Verwimp, 26725 (GOET) | JN631487 | JN631621 |  |  |  |
| *Scapania undulata* (L.) Dumort. | *Scapania undulata* (L.) Dumort. | Russia, Konstantinova, Bryo. Ross. Exs. 21 (GOET) | JN631488 | JN631622 |  |  |  |
| *Sphenolobus minutus* (Schreb. ex D.Crantz) Berggr. | *Sphenolobus minutus* (Schreb. ex D.Crantz) Berggr. | Russia, Arkhangelsk Province, Franz Josef Land, Ziegler Island, CA-19-29 (KPABG) | MT422255 | MT431399 |  |  |  |
| *Sphenolobus minutus* (Schreb. ex D.Crantz) Berggr. | *Sphenolobus minutus* (Schreb. ex D.Crantz) Berggr. | Svalbard, Spitsbergen, N.A. Konstantinova, K 68-1-06 (KPABG) | EU791789 | EU791667 |  |  |  |
| *Sphenolobus minutus* (Schreb. ex D.Crantz) Berggr. | *Sphenolobus minutus* (Schreb. ex D.Crantz) Berggr. | Russia, Kareliya Rep., V.A. Bakalin, 24.07.1998 | EU791790 |  |  |  |  |
